# Dental, Skeletal, and Soft Tissue Changes after Bone-Borne Surgically Assisted Rapid Maxillary Expansion: A Systematic Review and Meta-Analysis

**DOI:** 10.3390/dj11060143

**Published:** 2023-06-01

**Authors:** Christina Sekertzi, Maria-Marina Koukouviti, Athina Chatzigianni, Olga-Elpis Kolokitha

**Affiliations:** 1 Private Practice, 54636 Thessaloniki, Greece; christine.sekertzi@gmail.com (C.S.); markoukouv@gmail.com (M.-M.K.); 2 Department of Orthodontics, Faculty of Dentistry, School of Health Sciences, Aristotle University of Thessaloniki, 54124 Thessaloniki, Greece; okolok@dent.auth.gr

**Keywords:** bone-borne, surgically assisted, rapid maxillary expansion, SARME, SARPE, transverse deficiency

## Abstract

(1) Background: For non-growing patients with marked transverse maxillary deficiency, bone-borne surgically assisted rapid maxillary expansion (SARME) has been proposed as an effective treatment option. Objective: To evaluate the dental, skeletal, and soft tissue changes following bone-borne SARME. (2) Methods: An unrestricted systematic electronic search of six databases, supplemented by manual searches, was performed up to April 2023. The eligibility criteria included prospective/retrospective clinical studies with outcomes pertaining to objective measurements of dental/skeletal/soft tissue effects of bone-borne SARME in healthy patients. (3) Results: Overall, 27 studies satisfied the inclusion criteria. The risk of bias of the non-randomized trials ranged between moderate (20) and serious (4). For the two RCTs, there were some concerns of bias. Trials with outcomes measured at the same landmarks within the scope of the prespecified timeframe were deemed eligible for quantitative synthesis. Eventually, five trials were included in the meta-analysis. SARME was associated with a statistically significant lengthening of the dental arch perimeter immediately after expansion, along with a marginally significant decrease in palatal depth during the post-SARME retention period. Post-treatment SNA values exhibited no statistically significant change. (4) Conclusion: Current evidence indicates that bone-borne SARME constitutes an effective treatment option for adult patients with maxillary transverse deficiency. Further long-term randomized clinical trials with robust methodology, large sample sizes, and 3D evaluation of the outcomes are needed.

## 1. Introduction

Transverse maxillary discrepancies are a significant contributor to the emergence of orthodontic problems and represent a rather frequent issue faced during orthodontic treatment planning. The irregularities observed in these patients include lateral cross bites, narrow palatal vaults, increased buccal corridors, and dental crowding [1].

A variety of orthopedic and orthodontic techniques are available for transverse correction, particularly in young, growing patients. Nevertheless, in non-growing adolescents and adults, this particular challenge is more difficult to overcome, owing to the increased skeletal resistance observed post-ossification of the midpalatal suture [2]. Surgically assisted rapid maxillary expansion (SARME) has been introduced as a procedure aiming to address the treatment of marked transverse maxillary deficiencies in skeletally mature patients, with the goal of providing skeletal expansion with minimal dental effects [3]. Treatment with SARME entails a multi-disciplinary approach, which requires close collaboration between the orthodontic specialist and the oral-maxillofacial surgeon who will perform the orthognathic procedure [3].

SARME usually involves osteotomies akin to the Le Fort I technique, along with a mid-palatal suture disjunction, with or without pterygomaxillary disjunction (PMD). Subsequently, a palatal distractor is inserted in the patient’s oral cavity. This device can be attached to the posterior teeth of the patient (tooth-borne distractor) or directly to the maxilla (bone-borne distractor), or alternatively using a combination of both anchorage options (hybrid distractor) [4,5]. Previous studies have reported an array of approaches with regards to the surgical technique of choice used for the maxillary expander, the necessity for overcorrection, as well as the risk of relapse [5,6,7].

The effects of rapid maxillary expansion on hard and soft facial tissues have been investigated in individual studies via dental models, radiographic imaging, and, more recently, 3D facial scanners [8,9,10]. Several systematic reviews have assessed the differences between the outcomes of rapid maxillary expansion with tooth-borne and bone-borne devices, with results varying from no difference to the superiority of either method as far as different parameters are concerned [11,12,13]. However, limited information from individual studies is available on a meta-analytical level with regards to the evidence-based effects of bone-borne SARME, in terms of the objective measurements of the treatment-induced dental, skeletal, and soft tissue changes. A systematic review by Vilani et al. (2012) [14] examined the long-term dental and skeletal changes in SARME patients without differentiating between tooth-borne and bone-borne devices in the syntheses and found moderate evidence of an increase in alveolar and interdental widths. Similarly, Bortolotti et al. (2020) [15], in their recent review of RCTs investigating the skeletal and dental SARME effects, pooled data regardless of anchorage type and reported a successful expansion in the transverse maxillary dimension. Evidence focusing exclusively on bone-borne SARME would provide a multi-faceted understanding of the expected outcomes of the modality, thus assisting orthodontists in treatment plaining and enhancing the patient selection process. The aim of this systematic review was to assess evidence from clinical studies on the dental, skeletal, and soft tissue changes following bone-borne surgically assisted rapid maxillary expansion (SARME) on orthodontic patients.

## 2. Materials and Methods

### 2.1. Protocol, Eligibility Criteria, and Literature Search

The protocol of the present systematic review and meta-analysis was a priori developed in adherence with the Preferred Reporting Items for Systematic Reviews and Meta-Analysis Protocols guidelines (PRISMA-P) and was registered in PROSPERO (CRD42021250742). The study was conducted and reported according to the Cochrane Handbook for Systematic Reviews of Interventions [16] and the Preferred Reporting Items for Systematic Reviews and Meta-Analysis statement (PRISMA) [17]. The eligibility criteria, based on the PICOS schema of the review, included randomized clinical trials (RCTs), as well as prospective and retrospective clinical studies focusing on bone-borne SARME, with outcomes pertaining to objective measurements of dental, skeletal, and soft tissue effects of bone-borne SARME in otherwise healthy patients with transverse maxillary deficiency. Participants with a history of systemic disease or previous orthodontic treatment were excluded. No exclusions based on age, type of malocclusion, or the specific type of skeletal anchorage appliance used were applied. The outcomes comprised differences between pre-intervention and post-intervention measurements of the dental, skeletal, and soft-tissue characteristics reported either immediately after expansion (short-term effects) or during the retention period (long-term effects: 3 or more months post-expansion, in accordance with another meta-analysis) [12].

An unrestricted systematic electronic search of six databases (MEDLINE via PubMed, Cochrane Library/CENTRAL, Web of Science, CRD/DARE, Scopus, and Virtual Health Library) was performed by two independent researchers (C.S. and M.M.K.) up to April 2023. The search strategy was adapted for each database (available in the Appendix A, Table A1). The electronic search was supplemented by manual search of trial registries (Clinicaltrials.gov, WHO ICTRP, and EU Clinical Trials Register), grey literature sources (Open Grey, ProQuest Dissertations, and Theses), reference lists of the eligible studies, and relevant reviews. No restrictions regarding status, language, or year of publication were imposed.

### 2.2. Study Selection, Data Extraction, and Risk of Bias Assessment

Titles and abstracts were screened for relevance against the pre-specified inclusion/exclusion criteria. Full texts of potentially eligible articles were acquired and further assessed.

For the collection of all relevant information from the selected studies, a customized data extraction form was developed and piloted prior to data extraction, so as to maximize its efficiency and amend possible errors or ambiguity. All procedures were conducted in duplicate by two independent reviewers (C.S. and M.M.K.) to minimize errors and potential bias. A few minor conflicts were resolved via arbitration by the two senior reviewers (A.C. and O.E.K.) serving as content experts. All reasons for the exclusion of full-text articles were recorded.

Data concerning all objective dental, skeletal, and soft tissue changes following bone-borne surgically assisted rapid maxillary expansion (SARME) on orthodontic patients were collected from studies measuring the relevant outcomes immediately after expansion (IA Exp (T2)) and/or in retention (3 months or more after expansion (Τ3)). Studies that presented only comparisons of mean or median measures were excluded from the analysis owing to our inability to define the absolute number of patients with changes in outcomes in the groups. In cases of studies investigating the effects of different orthodontic modalities, only the data pertaining to the SARME arm were extracted.

Risk of bias assessment was performed at study level, using the revised Cochrane risk-of-bias tool for randomized trials (ROB 2) [18] and the ROBINS-I tool (risk of bias in non-randomized studies of interventions) [19], for the eligible randomized and non-randomized studies, respectively. The bias judgements were made taking into account the lack of a separate comparator group (patients’ values post-intervention served as comparators). The risk of bias was assessed in duplicate (C.S. and M.M.K.), with any disagreements resolved via the above-mentioned process.

### 2.3. Data Synthesis

Data were considered eligible for pooling on the condition that an outcome was similarly measured and reported in at least two studies. A two-step multivariate random-effects meta-analysis for longitudinal clinical trials using mixed-effects models was performed. Owing to the discrepancy of the data, to match time margins of follow-up between controlled and non-controlled clinical trials, raw mean change was considered a more appropriate effect size in statistical analysis, instead of standardized mean difference (as initially planned in the PROSPERO protocol). In the case of eligible studies with missing or incomplete outcome data, those were either calculated from existing data, if possible, or a request was extended to the original investigators via email. For the primary outcomes, the effect sizes were reported as raw mean changes with their 95% confidence intervals (CIs). The data were synthesized and analyzed using the review writing software RevMan 5.3 (Nordic Cochrane Center, Copenhagen, Denmark). Meta-regression analysis was performed with R version 3.5.0., using the package “metafor” and the random effects model. *p*-values were two-tailed and were considered significant at a 5% significance level. Statistical heterogeneity between studies reporting the same outcomes was investigated via visual inspection of forest plots, the chi-squared test, and the inconsistency index (I^2^) with 95% UI (I^2^ > 75% indicating considerable heterogeneity, demanding further investigation) [20]. The level of significance was set at α = 0.05, except for the test of heterogeneity, where a value of 0.10 was implemented owing to low power.

Publication bias was investigated through visual inspection of funnel plots for asymmetry. According to the study protocol, several sensitivity and subgroup analyses were planned in order to test the robustness of the results and to investigate possible sources of heterogeneity, respectively.

The overall strength of evidence pertaining to each primary outcome was assessed in accordance with the GRADE (Grades of Recommendation, Assessment, Development, and Evaluation) process and the corresponding Summary of Findings tables was constructed [21].

## 3. Results

### 3.1. Study Selection

**Figure 1:**
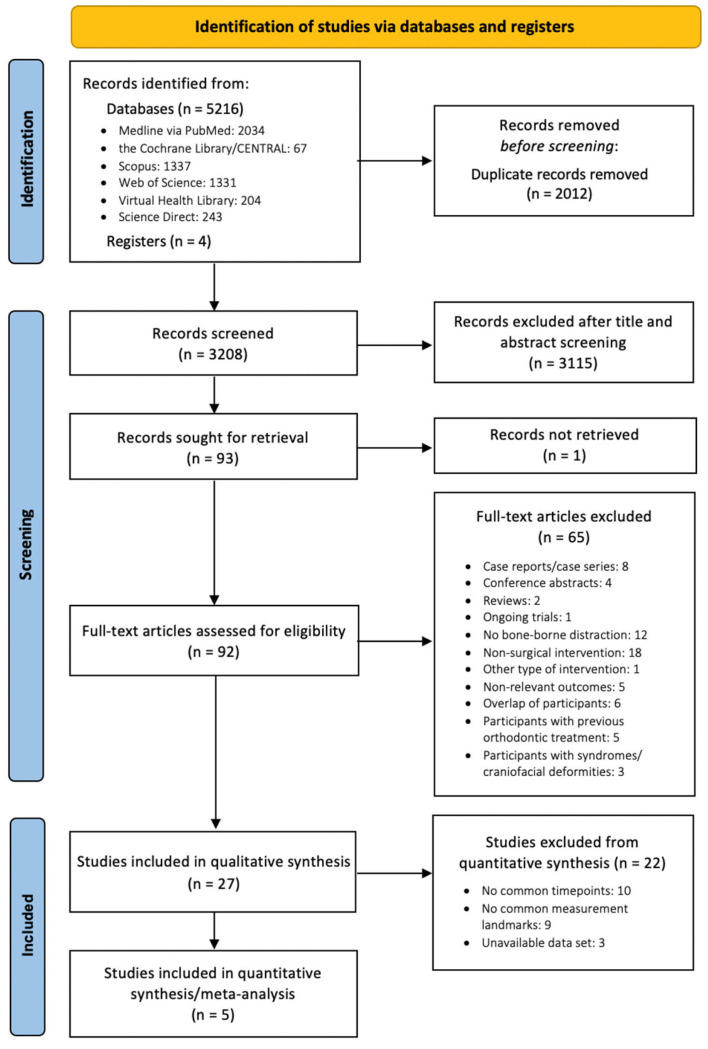
PRISMA 2020 flow diagram for study selection.

A total of 5216 articles were retrieved from the electronic databases, while 4 additional articles were identified manually (Figure 1). With the removal of the duplicates, 3208 records remained, and after screening of titles and abstracts, the full-text manuscripts of 92 articles were reviewed against the inclusion criteria. Eventually, a total of 27 publications with 621 patients were eligible for qualitative synthesis [4,22,23,24,25,26,27,28,29,30,31,32,33,34,35,36,37,38,39,40,41,42,43,44,45,46,47]. For the quantitative synthesis, we utilized a predefined timeframe for the short-term (immediately after expansion) and long-term outcomes (retention period: at least 3 months post expansion). Only trials that reported outcomes measured at the same landmarks and within the scope of the predefined timeframes were considered eligible for the meta-analytic process. Ultimately, eight trials satisfied these criteria [4,24,25,27,29,42,44,45], but four of them did not report their outcomes in variables suitable for data synthesis. Out of the four trial authors who were contacted for further access to the original dataset [4,27,42,44], only one responded [42]. Thus, five studies [24,25,29,42,45] with 233 patients were successfully included in the quantitative meta-analyses.

### 3.2. Study Characteristics

Out of the 27 included studies (Table 1), 2 were RCTs, 11 were prospective studies, 10 were retrospective studies, and 4 included both prospective and retrospective cohorts. All trials had been published as journal papers between 2001 and 2022. In some studies, pterygomaxillary disjunction (PMD) was also performed as part of the SARME intervention. Regarding the different designs of appliances used (distractor type), most studies used the (1) Transpalatal Distractor (TPD) (SurgiTec NV, Bruges, Belgium), followed by the (2) RPE^®^ expander (KLS Martin, Tuttligen, Germany) and the (3) MWD (Normed, Tuttlingen, Germany) distractor. The trials used different RME activation protocols, which ranged from 0.33 mm/day up to 1 mm/day, with the most common among them being the activation protocol of 1 mm/day. The mean age of patients varied between 16.9 and 30.2 years. As far as time points and follow ups are concerned, the measurements were obtained throughout different time periods, starting from immediately post-expansion, during the post-consolidation period, and lasting up to approximately 29 months post-operatively. The varied outcomes were measured via dental casts, CTs, lateral and posteroanterior (PA) cephalograms, and cone beam computerized tomography (CBCT), while in some studies, they were digitized and analyzed through 3D formats (list of types of outcomes available in the Appendix A, Table A2). The detailed tables of the characteris-tics and results of the eligible studies are available in the Supplementary Materials.

### 3.3. Risk of Bias within Studies

The risk of bias of the non-randomized trials included in the qualitative synthesis ranged between moderate (20 trials) and serious (4 trials), while for one trial, there was no adequate information for a bias judgment. The most serious concerns of bias were introduced by the probable existence of confounding factors, followed by the retrospective selection of participants, the lack of blinding of the outcome assessors, and missing data issues that were inadequately addressed in the data analysis (Table 2). For the included RCTs, there were some concerns of bias, mainly owing to the lack of blinding and issues with the randomization process (Table 3).

### 3.4. Results from Individual Studies

The majority of the included trials reported favorable results after bone-borne SARME, including a substantial increase in the arch perimeter [24,28,41] and a significant maxillary expansion at the intercanine, interpremolar, and intermolar level, as measured at the alveolar process, the gingival margin, between dental cups, or interapically [4,24,26,28,29,41,45]. In greater detail, one of the earliest studies investigating maxillary outcomes of bone-borne SARME by Pinto et al. (2001) found a post-operative relative increase in the arch perimeter of 10.5 ± 4.6% [41]. According to measurements derived from the selected cast landmarks (i.e., the most lingual points at the gingival margin), the corresponding expansion observed at the canine, first premolar, and first molar areas amounted to 35.7 ± 17%, 31.7 ± 14%, and 20.4 ± 8.7%, respectively [41]. Asscherickx et al. (2016), at 10 weeks post-expansion, reported a 6.1 ± 0.74 (SE) mm increase in arch perimeter, as well as increases of 5.7 ± 0.48 (SE) mm, 5.7 ± 0.38 (SE) mm, and 5.4 ± 0.42 (SE) mm in the distances between maxillary canines, premolars, and molars, respectively [24]. All distances were measured at the most lingual point of the gingival margin of the teeth [24]. A different study by Kunz et al. (2016), studying the outcomes of bone-borne distraction in 16 patients, used the distance between the palatal sulcus points of canines, premolars, and molars to report a progressive anteroposterior increase in the observed expansion, in both absolute and relative values (canines: 4.43 ± 2.21 mm, 19.72 ± 9.84%; first premolars: 4.56 ± 2.27 mm, 18.35 ± 9.13%; first molars: 3.53 ± 1.83 mm, 10.93 ± 5.67%) [29]. Barone et al. (2020) provided measurements from dental impressions at a 1-year postoperative follow-up [4]. In the bone-borne group, both interdental and intergingival distances were documented for canines (interdental: 2.18 ± 1.30 mm, intergingival: 2.21 ± 1.60 mm), premolars (interdental: 4.89 ± 3.05 mm, intergingival: 4.35 ± 2.86 mm), and molars (interdental: 3.22 ± 3.56 mm, intergingival: 2.66 ± 2.73 mm) [4], indicating an increase in all six variables. As far as the mandible was concerned, only one eligible article reported relevant outcomes, pointing to an increase in the dental show (2.5 ± 2.1 mm) and an inferoposterior repositioning of the chin (1.5 ± 2.2 mm posterior displacement) [46].

Transpalatal distraction was likely to cause a V-shaped pattern of expansion (larger in the anterior area and bilaterally asymmetrical on occasion) along with a slight downward maxilla movement [27,29,44]. This may be attributed to the new center of resistance being placed more posteriorly, which, in order to achieve a more parallel expansion, could be counteracted by the surgical release of the pterygoid plates [24]. SARME with bone-borne distractors could lead to the dropping and protrusion of the maxilla and its anterior movement may reflect the surgical procedure followed [25].

In some cases, the angulation of canines, premolars, and molars was also slightly altered, through buccal tipping [24]. However, Laudemann et al. (2011) [34] observed the presence of inward tipping in patients with no PMD and explained it as a compensation mechanism to the buccal tipping of the maxillary segments. Generally, with bone-borne appliances, tipping was observed to a limited extent. The effect on bone remodeling was minimal and mainly recorded in the area of the molars [30,31]. Petrick et al. (2011) [40] concluded that the midpalatal suture achieved up to ¾ of its pre-treatment density 7 months after SARME. A retention period of 6 months or more was suggested to avoid relapse [24,40].

The reported soft tissue changes included a posterior repositioning and decreased thickness of the upper lip and an increased projection of the cheek area [36]. In the nose area, Wallner et al. (2022) [45], using CBCT superimposed images of 91 patients, measuring a significant increase in both alar nasal width (1.2 ± 1.1 mm) and alar nasal base width (2.1 ± 1.2 mm), while the nose tip height remained unaffected. Conversely, Aras et al. (2017) [23] found that bone-borne SARME did not affect the soft tissue profile of the patients.

The main advantages of the procedure comprise minimal intraoperative complications, less vestibular bone resorption, and limited dental tipping [29,32]. Furthermore, bone-borne appliances allow for the possibility of an ongoing orthodontic treatment alongside the retention period, which may result in a reduced total treatment time [43]. However, the procedure involves increased expenses accompanied by an additional operation under local anesthesia for the removal of the appliance [43], while one study [42] also reported frequent mechanical failure.

With regards to the immediate outcomes post-expansion, the RCT of Koudstaal et al. (2009) [28] indicated an increase in the intercanine (6 ± 3.4 mm), interpremolar (7 ± 3.1 mm), and intermolar width (5.2 ± 3.4 mm). The different maxillary widths were measured on dental casts from the buccal cusps of the corresponding teeth. In addition, even though a slight relapse was observed after the 12-month follow-up, the net gain remained significant for all three measurements (intercanine: 4.7 ± 3.2 mm; interpremolar: 7.0 ± 3.5 mm; intermolar: 4.6 ± 3.1 mm). The overall increase in the arch perimeter was significant (6.0 ± 5.8). The palatal depth at premolar level decreased after 12 months at the premolar (–0.1 ± 2.1 mm) and molar level (–0.1 ± 2.1 mm). The palatal width at premolar level showed an increase of 2.9 mm (± 2.2) and, at molar level, an increase of 2.6 mm (± 2.5). It is worth noting that, in the large retrospective cohort of Wallner et al. (2022) [45], no significant decrease in the vertical palate height at first molars could be observed (−0.1 ± 1, *p* = 0.3082).

In the RCT of Zandi et al. (2014) [47], the largest percentage of expansion was observed in the dental arch, followed by the palatal bone, while the pattern of expansion was parallel posteroanterior. Furthermore, in both the first premolar and molar regions, the mean expansion gain at the interapical area was the same as that of the palatal bone area. Specifically, the palatal bone width showed an increase of 4.53 ± 2.02 mm at premolar level and an increase of 4.33 ± 1.23 mm at molar level. On the other hand, the interdental cusp distance increased by 6.73 ± 2.15 mm at premolar level and 6.53 ± 2.67 mm at molar level. Finally, the interdental root distance showed an increase of 4.40 ± 1.68 mm at premolar level and of 4.50 ± 1.83 mm at molar level.

### 3.5. Quantitative Synthesis

Trials with outcomes measured at the same landmarks within the scope of the prespecified timeframe for short-term and/or long-term effects were deemed eligible for quantitative synthesis. Nevertheless, out of the eight trials initially included, only four reported their outcomes via suitable variables [24,25,28,45] and, out of the other four, only one group of researchers [41] provided relevant data upon request. The additional dataset, however, was incomplete and could only be incorporated with regards to one quantitative variable (arch perimeter). Thus, five trials were eventually included in the meta-analyses (Table 4).

#### 3.5.1. Arch Perimeter

**Figure 2:**
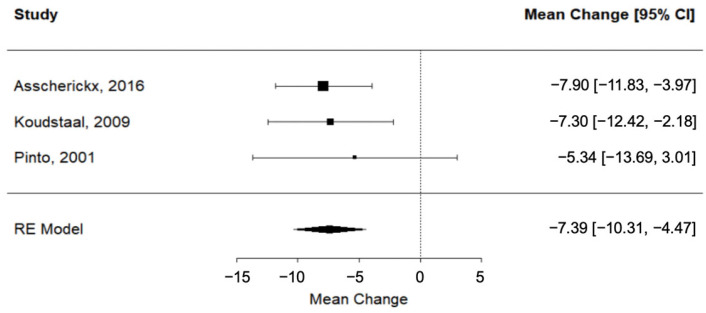
Forest plot for meta-analysis of arch perimeter from three trials [24,28,41].

According to evidence from three different trials [24,28,41], SARME was associated with a significant lengthening of the dental arch perimeter immediately after expansion (RC = −7.39; 95% CI = lower: −10.31, upper: −4.47; *p*-value < 0.001; I^2^ = 0%) (Figure 2).

#### 3.5.2. Palatal Depth

**Figure 3:**
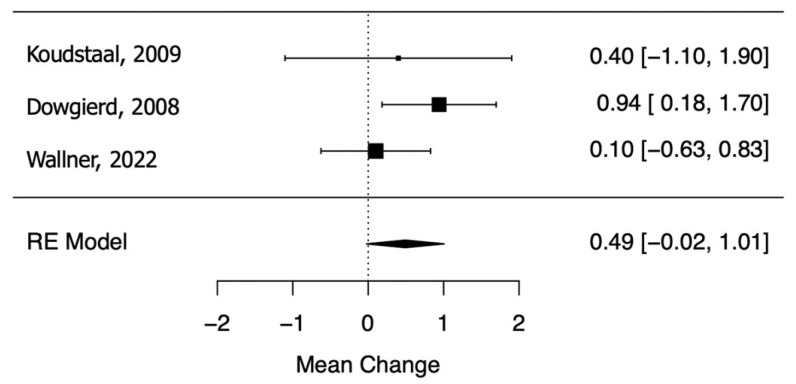
Forest plot for meta-analysis of palatal depth from three trials [25,28,45].

Dowgierd et al. (2018) [25], Koudstaal et al. (2009) [28], and Wallner et al. (2022) [45] provided evidence of a marginally significant decrease in palatal depth during the retention period after SARME (RC = 0.49; 95% CI = lower: −0.02, upper: 1.01; *p*-value = 0.06; I^2^ = 5.61%) (Figure 3).

#### 3.5.3. Radiograph Outcomes

**Figure 4:**
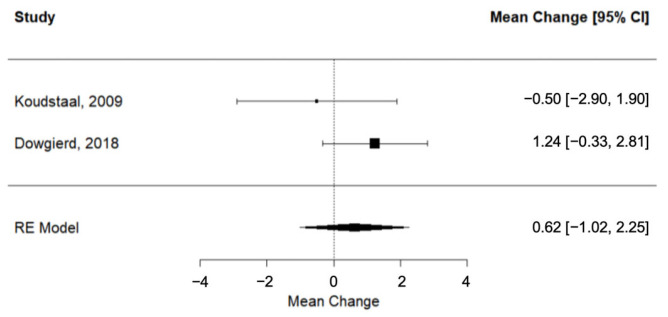
Forest plot for meta-analysis of SNA values from two trials [25,28].

Two trials [25,28] provided evidence on the observed differences in radiographic measurements during post-SARME retention. The pooled SNA outcome did not suggest a statistically significant change (SNA: RC = 0.62; 95% CI= lower: −1.02, upper: 2.25; *p*-value = 0.46; I^2^ = 29.33%) (Figure 4).

#### 3.5.4. Publication Bias

**Figure 5:**
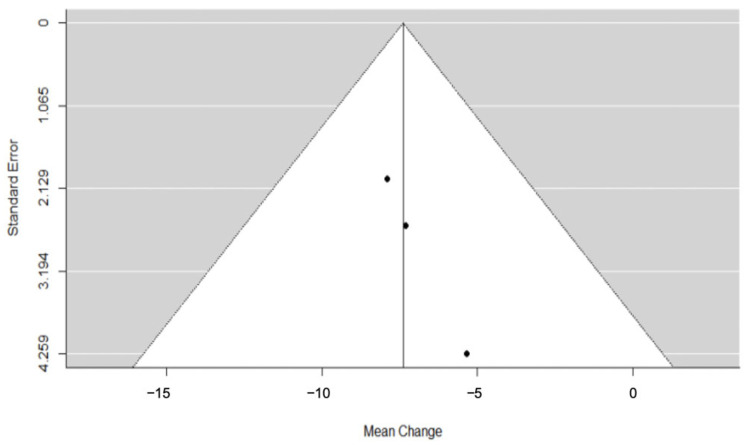
Funnel plot for meta-analysis of arch perimeter from three trials.

**Figure 6:**
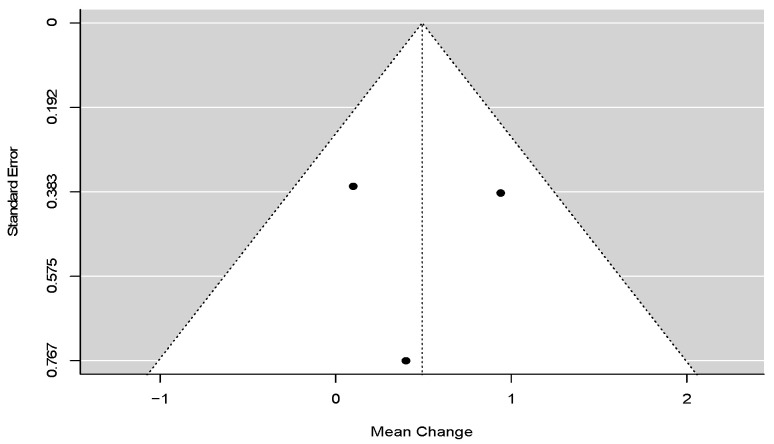
Funnel plot for meta-analysis of palatal depth from three trials.

**Figure 7:**
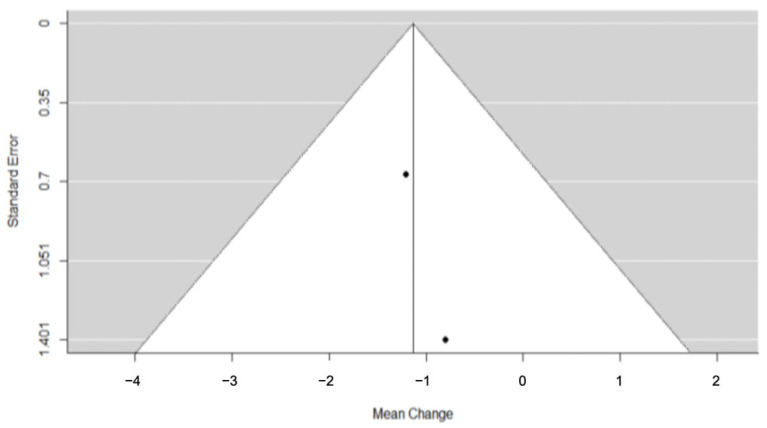
Funnel plot for meta-analysis of SNA from two trials.

Upon inspection, none of the resulting funnel plots demonstrated obvious signs of asymmetry that would suggest the existence of severe publication bias. Nonetheless, given the small number of the included studies per individual meta-analysis, these conclusions should be regarded with caution (Figure 5, Figure 6 and Figure 7).

#### 3.5.5. Sensitivity and Subgroup Analyses

The analyses planned to investigate the robustness of the results and the possible sources of heterogeneity, respectively, were ultimately proven infeasible owing to the small number of relevant trials available.

#### 3.5.6. Quality of Evidence

The quality of evidence for the meta-analyses was evaluated with the GRADE (Grading of Recommendations Assessment, Development, and Evaluation) approach. The domains’ risk of bias, directness of evidence, consistency and precision of results, and risk of publication bias were assessed for each outcome. The judgments for each outcome can be found in Table 5. The overall quality of evidence was deemed to be low.

GRADE Working Group grades of evidence

High certainty: We are very confident that the true effect lies close to that of the estimate of the effect.

Moderate certainty: We are moderately confident in the effect estimate: The true effect is likely to be close to the estimate of the effect, but there is a possibility that it is substantially different.

Low certainty: Our confidence in the effect estimate is limited: The true effect may be substantially different from the estimate of the effect.

Very low certainty: We have very little confidence in the effect estimate: The true effect is likely to be substantially different from the estimate of effect.

Explanations

- Downgraded by one point for some concerns of bias (with regards to the randomization process and confounding).
- Downgraded by one point for imprecision (small sample of participants).
- Downgraded by one point for some concerns of bias (with regards to the randomization process, confounding, and selection of participants).

## 4. Discussion

The aim of this systematic review was to assess the evidence available from clinical studies on the objective dental, skeletal, and soft tissue changes following bone-borne surgically assisted rapid maxillary expansion (SARME) in orthodontic patients. The comparisons were conducted without a control group; instead, individuals were analyzed at different time points to examine intervention outcomes. Overall, 27 studies satisfied the inclusion criteria of human clinical trials reporting outcomes for short-term and/or long-term effects due to SARME procedures and were included in the qualitative review. The risk of bias of the non-randomized trials included in the qualitative synthesis ranged between moderate (20 trials) and serious (4 trials), while for one trial, there was no adequate information for a bias judgment. For the two included RCTs, there were some concerns of bias, mainly due to the lack of blinding and issues with the randomization process. Trials with outcomes measured at the same landmarks within the scope of the prespecified timeframe for short-term and/or long-term effects were deemed eligible for further quantitative synthesis. Out of the eight trials initially selected, five trials [24,25,28,41,45] reported their outcomes using the same variables and were eventually included in the meta-analyses.

From the results of this meta-analysis, it can be inferred that SARME was associated with a significant lengthening of the dental arch perimeter immediately after expansion, as well as with a marginally significant decrease in palatal depth during the post-SARME retention period. The existing data with regards to observed changes in SNA values, which were obtained via radiographic measurements during the post-SARME retention period, did not denote any statistically significant changes. The present evidence indicates that SARME, as a treatment option for transverse deficiencies, may achieve some of the intended effects. However, these results should be viewed with caution owing to the small number of studies included in the analyses and the concerns of bias.

The present study is the first one to meta-analyze the available bibliographic evidence pertaining to changes observed in the measurements of the arch perimeter, the palatal depth, and the SNA angle after bone-borne SARME. Owing to the specificity of the meta-analytic data, no direct comparisons to other published papers regarding this particular method or other alterative interventions (e.g., bone-borne SARME) could be drawn. It could be noted that the review by Vilani et al. (2012) [14], which similarly investigated post-SARME changes in dental and skeletal structures using the metrics of patients at different time points as self-comparators, reported a significant long-term increase with regards to the maxillary alveolar width and to the interdental widths at the canine and molar level. Nevertheless, the results of their study were reported without differentiating between tooth-borne and bone-borne appliances. Bortolotti et al. (2020) [15], in their meta-analysis of RCTs regardless of type of anchorage, also found significant maxillary expansion on a skeletal and inter-molar level, with the latter being the main contributor to the overall effect. Even though the results from the individual studies included in the present systematic review likewise suggest that bone-borne SARME causes a significant increase in dental and skeletal width parameters, those data did not satisfy our criteria for pooling, as the researchers did not use the same landmarks or similar time-points in their measurements. It is worth mentioning that evidence from studies with multiple timepoints, even though they show some relapse of the gained expansion at their longest follow-ups, is not significant, thus suggesting that the overall effect of the modality is net positive and relatively stable [22,24,42].

The limited number and quality of the eligible studies yielded during this systematic review highlights the need for additional original research into the precise effects of SARME. Randomized controlled trials that meet high-quality standards could further establish the treatment scope of the modality and provide strong guidance to clinicians with regards to patient selection and expected results. Possible suggestions for future studies include the investigation of both short- and long-term SARME outcomes, with a specific focus on the effect of differentiated parameters, such as surgical techniques and the type of bone-borne distractor.

Among the strengths attributed to this meta-analysis can be considered the a priori registration protocol in PROSPERO, a scrupulous database and hand search, the strict inclusive and exclusive criteria, and the use of the GRADE approach to assess the quality of the evidence. However, several limitations also exist. First and foremost, there was no exclusion of non-randomized trials, leading to a greater extent of methodological limitations and bias. Several studies deemed eligible for qualitative synthesis reported insufficient control for confounding factors, less than optimal participant selection, and lack of blinding, thus introducing further concerns of possible bias. Furthermore, the varied measurement techniques used to assess skeletal, dental, and soft-tissue changes made it impossible to compare and synthesize all selected studies, which could have strengthened the quality of evidence. The limited number of included studies and heterogeneity in measurement techniques may introduce restrictions to the generalization of the results. Finally, subgroup analyses (including, e.g., different types of distractors, activation protocols, appliances used for retention, time period, and surgical techniques) could not be performed because of the small number of included studies.

## 5. Conclusions

According to the results of the current meta-analysis, there is low evidence that the dental arch perimeter immediately post-expansion exhibits a significant increase as a result of bone-borne SARME. Generally, the findings indicate that bone-borne SARME constitutes an effective treatment modality for adult patients with maxillary transverse deficiency, hence it may be used to successfully expand a constricted maxilla. Nevertheless, the restricted number of high-quality trials impedes drawing strong conclusions that could offer definite clinical guidance. Future long-term randomized controlled trials with a robust methodology and standardized protocols are needed to minimize bias and further elucidate the precise effects of SARME.

## Figures and Tables

**Table 1:** Characteristics and representative results of studies included in the qualitative synthesis.

Trial ID	Study Design	Pterygomaxillary Disjunction (PMD)	Distractor	Activation Time	Number of Patients	Mean Age—Sex (M/F)	Measuring Method	Timepoints	Selected Results (Mean Difference (SD), unless Otherwise Indicated)
Aras et al. (2010) [22]	prospective cohort	no	Transpalatal Distractor, SurgiTec NV (Bruges, Belgium)	1 mm/day (7–10 days)	11	27.3 — 5/6	CT	T1 (baseline) T2 (6.7 ± 2.3 months after surgical intervention, at expander removal)	*Canines* Bimaxillary width: **6.59** Bialveolar width: **7.37** *Molars* Bimaxillary width: **4.55** Bialveolar width: **5.95**
Aras et al. (2017) [23]	retrospective cohort	no	Transpalatal Distractor, SurgiTec NV (Bruges, Belgium)	1 mm/day (7–10 days)	16	27.4 — 9/7	lateral cephalometric radiographs	T1 (baseline) T2 (after expansion—before any further ortho treatment)	SNA: **0.63°** SNB: **−0.64°** ANPg: **1.07°** SNGoGn: **0.85°** Basic upper lip thickness: **−0.61**
Asscherickx et al. (2016) [24]	prospective cohort	no	Transpalatal Distractor, SurgiTec NV (Bruges, Belgium)	1 mm/3 days (8–22 days)	21	26.5 — 6/15	study casts, posteroanterior cephalograms	T1 (baseline) T2 (end of expansion) T3 (10 weeks after T2)	Intercanine width (T2): **6.6** (0.35) Intercanine width (relapse): −1.0 (0.38) Intermolar width (T2): **5.2** (0.36) Intermolar width (relapse): 0.1 (0.35) Arch perimeter (T2): **7.8** (0.47) Arch perimeter (relapse): **2.4** (0.39)
Barone et al. (2020) [4]	prospective cohort	no	RPE^®^ expander (KLS Martin, Tuttligen, Germany)	1 mm/day	12	NA — NA	virtual study models from dental casts	T1 (baseline) T2 (1 year post-operatively)	Interdental canine distance: 2.18 (1.30) Interdental premolar distance: 4.89 (3.05) Interdental molar distance: 3.22 (3.56) Intergingival canine distance: 2.21 (1.60) Intergingival premolar distance: 4.35 (2.86) Intergingival molar distance: 2.66 (2.73) *(no data on significance)*
Dowgierd et al. (2018) [25]	retrospective cohort	yes	Titamed Smile Distractor	approx. 0.6 mm/day	78	16.86 — NA	CBCT, lateral cephalograms	T1 (baseline) T2 (3 months post distraction)	*(pre- and post-treatment values)* ANB: −0.75 (4.47)/0.48 (4.04) SNA: 82.52 (5.69)/81.28 (4.19) SNB: 82.05 (5.23)/81.60 (5.06) S-PNS: 47.42 (4.69)/47.87 (4.48) N-ANS: 50.72 (4.24)/51.32 (4.45) Molar distance (dental): **36.94 (3.47)/41.77 (4.26)** Molar distance (alveolar): **31.44 (3.06)/36.25 (3.92)** Palatal height: 14.32 (2.41)/13.38 (2.67)
Hansen et al. (2007) [26]	prospective cohort	yes	Dresden distractor	0.96 mm/day (7 days)	12	25.3 — NA	CT	T1 (baseline) T2 (post expansion)	Midpalatal suture expansion—ANS: 3.00 (1.49) Midpalatal suture expansion–PNS: 0.97 (0.92) Alveolar process expansion—premolars: 5.55 (2.63) Alveolar process expansion—molars: 4.87 (2.44) Crown expansion—premolars: 6.07 (2.97) Crown expansion—molars: 5.71 (2.42) Root-apex expansion—premolars: 4.28 (2.99) Root-apex expansion—molars: 4.98 (2.28) Molar tipping (range): 1.1–2.6 Alveolar process tipping (range): 8–9.8
Huizinga et al. (2018) [27]	retrospective cohort	yes	TPD (Classic or All-in-one, Surgi-Tec, Sint-Denijs-Westrem, Belgium)	0.33–0.66 mm/day	20	24.5 — 12/8	CBCT	T1 (baseline) T2 (post expansion)	*lateral expansion in 5 directions [median (IQR)]* Inferior–anterior (right maxillary segment): 0.36 (−1.42, 3.29) Inferior–posterior (right m.s.): −0.03 (−0.54, 1.74) Superior–posterior (right m.s.): 0.10 (−0.45, 1.46) Anterior vs. posterior (anterior maxillary part): −0.53 (−1.40, 1.13) Caudal vs. cranial (caudal maxillary part): 1.51 (0.69, 1.93)
Koudstaal et al. (2009) [28]	RCT	no	Transpalatal Distractor, SurgiTec NV (Bruges, Belgiumor RPE^®^ expander (KLS Martin, Tuttligen, Germany)	1 mm/day	23 (25 randomized)	33 — 10/15	lateral and PA cephalograms, casts	T1 (baseline) T2 (post expansion) T3 (12 months after treatment)	Intercanine width (T2): **6** (3.4) Intercanine width (relapse): −1.3 (3.2) Intermolar width (T2): **5.2** (3.4) Intermolar width (relapse): −0.6 (1.5) Arch perimeter (T2): **7.3** (3.7) Arch perimeter (relapse): −1.3 (4.5) Palatal depth at molar (net change): **−0.4** (0.7) SNA (net change): 0.5 (1.3)
Kunz et al. (2016) [29]	retrospective cohort	yes (partial separation of pterygoid process)	Transpalatal Distractor, SurgiTec NV (Bruges, Belgium or RPE^®^ expander (KLS Martin, Tuttligen, Germany)	0.66 mm/day (2–3 weeks)	16	26.5 — 6–10	3D models dental casts	T1 (baseline) T2 (5.6 ± 3.5 months post distraction)	Transverse distance—canines: 4.43 (2.21) Transverse distance—first premolars: 4.56 (2.27) Transverse distance—second premolars: 4.18 (1.77) Transverse distance—first molars: 3.53 (1.83) Transverse distance—second molars: 2.72 (1.19)
Landes et al.(2009a) [30]	retrospective and prospective cohort	yes/no	MWD (Normed, Tuttlingen, Germany) or TPD (Surgi-Tec, Bruges, Belgium)	0.5–0.6 mm/day	8 (bipartite) 11 (tripartite)	NA — NA	3D-CT scans	T1 (baseline) T2 (post expansion)	Skeletal widening (skeletal)—Bipartite: −2.73 (2.24) Skeletal widening (skeletal)—Tripartite: −4.4 (3.59) Skeletal widening (alveolar)—Bipartite: −3.01 (2.14) Skeletal widening (alveolar)—Tripartite: −3.98 (2.39) Segmental inclination—Bipartite: −0.60 (2.95) Segmental inclination—Tripartite: 4.44 (2.38) Dental widening (tips)—Bipartite: −3.76 (2.18) Dental widening (tips)—Tripartite: −4.5 (4.42) Dental widening (buccal prominence)—Bipartite: −4.05 (2.99) Dental widening (buccal prominence)—Tripartite: −5.08 (5.29)
Landes et al.(2009b) [31]	retrospective and prospective cohort	yes/no	MWD (Normed, Tuttlingen, Germany) or TPD (Surgi-Tec, Bruges, Belgium)	0.5–0.6 mm/day	24 (50)	NA — NA	3D-CT scans	T1 (baseline) T2 (post expansion)	Skeletal widening (skeletal)—first premolars: 6.51 (3.19) Skeletal widening (skeletal)—first molars: 3.19 (1.87) Skeletal widening (alveolar)—first premolars: 7.16 (2.91) Skeletal widening (alveolar)—first molars: 5.07 (1.63) Dental widening (tips)—first premolars: 7.51 (3.3) Dental widening (tips)—first molars: 7.12 (2.29) Dental widening (buccal prominence)—first premolars: 7.30 (2.49) Dental widening (buccal prominence)—first molars: 6.84 (1.97)
Laudemann et al. (2009) [32]	retrospective and prospective cohort	yes/no	MWD (Normed, Tuttlingen, Germany) or TPD (Surgi-Tec, Bruges, Belgium)	0.5–0.6 mm/day	24	NA — NA	3D-CT scans	T1 (baseline) T2 (4–26 weeks post expansion)	Transverse widening (skeletal—with PMD): −4.50 (3.91) Transverse widening (skeletal—no PMD): −2.61 (2.43) Transverse widening (alveolar—with PMD): −3.00 (2.44) Transverse widening (alveolar—no PMD): −3.52 (2.69) Segmental inclination (with PMD): 1.50 (2.86) Segmental inclination (no PMD): −0.91 (1.89)
Laudemann et al. (2010) [33]	retrospective cohort	yes/no	MWD (Normed, Tuttlingen, Germany) or TPD (Surgi-Tec, Bruges, Belgium)	0.5—0.6 mm/day	18	NA — NA	3D scanned cast models	T1 (baseline) T2 (20.5 ± 1.34 months post-expansion)	Transverse skeletal widening (gingival margin): 1.61 (1.76) Transverse skeletal widening (cusp tips): 0.99 (3.28) Dental tipping—canines: −0.21 (0.95) Dental tipping—first premolar: −0.06 (1.27) Dental tipping—first molar: 0.22 (1.01)
Laudemann et al. (2011) [34]	retrospective and prospective cohort	yes/no	MWD (Normed, Tuttlingen, Germany) or TPD (Surgi-Tec, Bruges, Belgium)	0.5–0.6 mm/day	25	NA — NA	3D-CT scans	T1 (baseline) T2 (2.87 ± 1.59 months post-expansion)	Transverse widening (skeletal) (<20 years old—with PMD): −4.72 (5.34) Transverse widening (skeletal) (>20 years old—with PMD): −4.03 (1.73) Transverse widening (skeletal) (<20 years old—no PMD): −2.77 (2.09) Transverse widening (skeletal) (>20 years old— no PMD): −3.01 (2.83) Transverse widening (alveolar) (<20 years old—with PMD): −2.50 (3.07) Transverse widening (alveolar) (>20 years old—with PMD): −3.18 (2.16) Transverse widening (alveolar) (<20 years old—no PMD): −3.58 (2.32) Transverse widening (alveolar) (>20 years old— no PMD): −4.48 (1.33)
Matteini et al. (2001) [35]	prospective cohort	yes	TPD	0.33 mm/day	20	20 — 8/12	Models	T1 (before surgery) T2 (post expansion, 2–3 weeks later)	Transverse expansion—canines: 29.9% (14.1) Transverse expansion—first premolars: 28.3% (11.6) Transverse expansion—first molars: 20.8% (7.2)
Nada et al. (2012) [36]	prospective cohort	yes	TPD (Surgi-Tec, Bruges, Belgium)	1 mm/day	17	29.4 — NA	3D CBCT models	T1 (baseline), T2 (22 ± 7 months after completion of pre-surgical orthodontic treatment and prior to second orthognathic intervention)	Interocclusal expansion—first premolars: 6.24 (2.3) Interocclusal expansion—first molars: 7.14 (3.7) Interapical expansion—first premolars: 5.2 (3.2) Interapical expansion—first molars: 4.6 (3)
Nada et al. (2013) [37]	prospective cohort	yes	TPD (Surgi-Tec, Bruges, Belgium)	1 mm/day	15	30 — 7/8	3D CBCT models	T1 (baseline) T2 (22 ± 7 months after completion of pre-surgical orthodontic treatment)	Lip, middle segment: −1.6 (1.9) Lip, right segment: −0.45 (2.3) Lip, left segment: −0.48 (1.8) Maxilla, middle segment: −1.12 (1.5) Maxilla, right segment: 1.97 (0.9) Maxilla, left segment: 1.82 (0.9)
Nikolaev et al. (2017) [38]	retrospective cohort	NA	NA	NA	21	NA — NA	CBCT	T1 (baseline) T2 (day of the expander removal)	Interapical distance first premolars: 3.1 (0.4) Interapical distance molars: 2.3 (0.3) Intercoronal distance first premolars: 4.8 (0.5) Intercoronal distance molars: 4.1 (0.4)
Parhiz et al. (2011) [39]	retrospective cohort	yes	TPD (SurgiTec, Bruges, Belgium)	0.33 mm/day	50	26 — 20/30	posteroanterior (PA) and lateral cephalograms, panoramic and periapical radiographs, intraoral and extraoral photographs, study models	T1 (baseline) T2 (20 ± 9 months)	SNA: 1.60 (2.57) SNB: 0.46 (2.61) ANB: 1.06 (2.00) U1-SN: −4.82 (8.94) U1-PP: −3.86 (8.56)
Petrick et al. (2011) [40]	prospective cohort	no	Dresden Distractor (DD; ITU, Dresden, Germany)	1 mm/day (8 days)	16	24.5 — 7/9	CT	T1 (baseline) T2 (on average 7.01 months after SARME)	Midpalatal suture width—anterior: 2.30/168% Midpalatal suture width—median: 1.12/140% Midpalatal suture width—posterior: 0.26/111%
Pinto et al. (2001) [41]	prospective cohort	no	TPD (Surgi-Tec, Bruges, Belgium)	0.33 mm/day	20	21.5 — 9/11	Digital photographs of the models (dental casts)	T1 (baseline) T2 (end of expansion)	Expansion—first premolars (%): 31.7 (14) Expansion—first molars (%): 20.4 (8.7) Arch periphery gain: 10.5 (4.6) Angulation of first premolars: −8.3 (9.6) Angulation of first molars: 0.9 (9.9)
Ploder et al. (2021) [42]	retrospective cohort	yes	TPD (Surgi-Tec, Bruges, Belgium) or OMI appliance (Micro-4 Hyrax appliance (MICRO4), Tiger Dental, Bregenz, Austria)	TPD: 1 mm/day (12.6 ± 5.8 days) OMI: 0.51 mm/day (12.5 ± 1.3 days)	12 (TPD) 13 (OMI)	TPD: 24.8 OMI: 36.1 — TPD: 5/7 OMI: 4/9	Cast models Panoramic radiographs	T1 (before surgery, 1–7 days) T2 (after consolidation period, 8–10 weeks) T3 (1 year after surgery, range 10–14 months)	*Tooth level TPD* Overall expansion: 5.22 mm (1.72) Relapse: 0.76 mm (1.37) Canines: 4.76 mm (3.00) Relapse: 1.33 mm (1.25) First molars: 4.91 mm (2.64) Relapse: 0.06 mm (1.63) *Bone level TPD* Overall expansion: 4.66 mm (2.03) Relapse: 0.71 mm (0.96) Canines: 4.38 mm (1.57) Relapse: 0.85 mm (1.59) First molars: 4.89 mm (3.08) Relapse: 0.50 mm (1.55)
Seeberger et al. (2015) [43]	prospective cohort	yes	Titamed Uni-Smile Distractor (Wervik, Belgium)	0.5 mm/day	19	22 — 8/11	CBCT	T1 (1 month before) T2 (3 months after surgery)	*(median (IQR))* Premolar crown width: 4.6 (3.4) Premolar apex width: 3.3 (3.1) Molar crown width: 3.40 (2.4) Molar apex width: 3.20 (2.8)
Tausche et al. (2007) [44]	prospective cohort	no	DD—Dresden Distractor	over- compensation of 0.5–1 mm (8–10 days)	17	28.8 — 6/11	CT	T1 (before) T2 (6 months after insertion of DD)	Intercoronal width—first premolars: **6.72** (2.58) Intercoronal width—first molars: **6.44** (1.92) Interapical width—first premolars: **5.79** (2.65) Interapical width—first molars: **6.53** (2.07)
Wallner et al. (2022) [45]	retrospective cohort	yes	Titamed SMILE 3-distractor (Kontich, Belgium)	0.5 mm/day (0.25 mm twice a day) for 14 days	91	20 — 33/58	superimposed CBCT images	T1 (baseline) T2 (approx. 1 year postoperatively)	Vertical palate height at first molars: −0.1 (1.0) Alveolar width at canines: **4.8** (1.8) Alveolar width at first molars: **3.8** (1.4)
Xi et al. (2017) [46]	retrospective cohort	yes (only in cases of asymmetric mobility)	TPD (UNI-Smile distractor, Titamed, Kontich, Belgium).	1 mm/day (consolidation period of 8–10 weeks)	78 with hyrax group	30.2 — 22/56	3D cephalometric reference frame from CBCT	T1 (baseline) T2 (20.3 ± 6.2 months)	Dental show: 2.5 (2.1) Mandibular plane angle: 1.1 (1.1) Vertical changes at A-point: 1.6 (2.3) Vertical changes at pogonion: 1.8 (1.8) Anterior maxillary expansion: 1.8 (1.0) Posterior maxillary expansion: 2.6 (1.8) Chin advancement: −1.5 (2.2)
Zandi et al. (2014) [47]	RCT	yes	TPD (SurgiTec, Bruges, Belgium)	0.5–0.6 mm/day up to an overexpansion of 2–3 mm	15	19.4 — 5/10	CBCT	T1 (before operation) T2 (immediately after consolidation period)	Palatal bone width: 4.33 (1.23) Interdental cusp distance—first premolars: 6.73 (2.15) Interdental cusp distance—first molars: 6.53 (2.67) Interapical distance—first premolars: 4.4 (1.68) Interapical distance—first molars: 4.5 (1.83)

CBCT: cone beam computed tomography; CT: computed tomography; NA: not available; PA: posteroanterior; RCT: randomized controlled trial.

**Table 2:** Risk of bias assessment for the eligible non-randomized trials (ROBINS-I).

Trial ID	1. Confounding	2. Selection of Participants for the Study	3. Classification of Interventions	4. Deviations from Intended Interventions	5. Missing Data	6. Measurement of Outcomes	7. Selection of the Reported Result	*Overall*
Aras et al. (2010) [22]	+	++	++	++	++	+	++	+
Aras et al. (2017) [23]	+	+	++	++	++	+	++	+
Asscherickx et al. (2016) [24]	+	++	++	++	++	++	++	+
Barone et al. (2020) [4]	+	++	++	++	++	+	++	+
Dowgierd et al. (2018) [25]	+	+	++	++	++	+	++	+
Hansen et al. (2007) [26]	+	+	++	++	++	+	++	+
Huizinga et al. (2018) [27]	+	-	++	++	+	+	++	-
Kunz et al. (2016) [29]	+	+	++	++	++	+	++	+
Landes et al. (2009a) [30]	+	+	++	++	+	++	++	+
Landes et al. (2009b) [31]	+	+	++	++	+	++	++	+
Laudemann et al. (2009) [32]	+	+	++	++	+	++	++	+
Laudemann et al. (2010) [33]	+	+	++	++	+	++	++	+
Laudemann et al. (2011) [34]	+	+	++	++	+	++	++	+
Matteini et al. (2001) [35]	+	++	++	++	++	+	++	+
Nada et al. (2012) [36]	+	++	++	++	++	++	++	+
Nada et al. (2013) [37]	+	++	++	++	++	++	++	+
Nikolaev et al. (2017) [38]	+	?	++	++	++	+	++	?
Parhiz et al. (2011) [39]	+	+	++	++	++	+	++	+
Petrick et al. (2011) [40]	+	++	++	++	++	++	++	+
Pinto et al. (2001) [41]	+	++	++	++	++	++	++	+
Ploder et al. (2021) [42]	+	+	++	-	++	+	++	-
Seeberger et al. (2015) [43]	+	+	++	++	++	+	++	+
Tausche et al. (2007) [44]	+	-	++	++	++	+	++	-
Wallner et al. (2022) [45]	+	+	++	++	++	+	++	+
Xi et al. (2017) [46]	+	-	++	++	++	+	++	-

++ low risk of bias; + moderate risk of bias; - serious risk of bias; ? no information.

**Table 3:** Risk of bias assessment for the eligible randomized trials (RoB 2).

Trial ID	1. Randomization Process	2. Deviations from Intended Interventions	3. Missing Outcome Data	4. Measurement of the Outcome	5. Selection of the Reported Result	Overall
Koudstaal et al. (2009) [28]	*	+	+	+	*	*
Zandi et al. (2014) [47]	+	*	*	+	*	*

+ low risk of bias; * some concerns.

**Table 4:** Results of random effects meta-analyses performed for relevant outcomes after SARME.

Outcome	No of Trials	Time Point	RC	95% CI	SE	P	I^2^ (95% CI)	t^2^ (95% CI)	P (Q)
*Arch perimeter*	3	IA Exp	−7.39	−10.31, −4.47	1.49	**<0.001**	0%	0	0.86
*Palatal depth*	3	Ret	0.49	−0.02, 1.01	0.26	**0.06**	5.61%	0.01	2.48
*SNA*	2	Ret	0.62	−1.02, 2.25	0.83	0.46	29.33%	0.44	0.23

IA Exp: immediately after expansion; RC: raw change; Ret: retention period.

**Table 5:** GRADE summary of findings.

Outcomes	Anticipated Absolute Effects (95% CI)		No of Participants (Studies)	Certainty of the Evidence (Grade)	Comments
	Risk before SARME	Risk after SARME			
Arch perimeter assessed with dental casts Follow-up: range 2 weeks to 12 months	The mean arch perimeter was 69.92 mm	RC −7.39 (increase) (−10.31 to −4.47)	64 (1 RCT, 2 observational studies)	⨁⨁◯◯ Low [24,28,41] ^a, b^	The evidence suggests that treatment with SARME increases the arch perimeter.
Palatal depth assessed with CBCT, dental casts Follow-up: range 3 months to 12 months	The mean palatal depth was **18.77** mm	RC **0.49** **(decrease)** (−0.02 to 1.01)	192 (1 RCT, 2 observational studies)	⨁⨁◯◯ Low [24,28,45] ^b, c^	The evidence suggests that treatment with SARME may result in a slight reduction in palatal depth.
SNA assessed with CBCT, lateral cephalograms Follow-up: range 3 months to 12 months	The mean SNA was **81.88°**	RC **0.62** **(decrease)** (−1.02 to 2.25)	101 (1 RCT, 1 observational study)	⨁⨁◯◯ Low [25,28] ^b, c^	The evidence suggests that treatment with SARME may result in little to no difference in SNA.

CI: confidence interval; RC: raw change; RCT: randomized controlled trial.

## Data Availability

The data that support the findings of this study are available from the authors upon request.

## Supplementary Materials

The following supporting information can be downloaded at: https://www.mdpi.com/article/10.3390/dj11060143/s1.

## Appendix A

**Table A1:** Search strategy used in each database.

Database	Search Strategy
MEDLINE via Pubmed	(surg* OR “surgically-assisted”) AND ((palat* OR maxill* OR “upper jaw” OR “upper arch” OR transpalatal) AND (expansion OR expand* OR distract* OR enlargement)) AND (miniscrew* OR “mini-screw*” OR “micro-screw*” OR “mini-implant*” OR “micro-implant*” OR microimplant* OR “skeletal anchor*” OR “bone-borne” OR “bone borne” OR “bone-anchored” OR “bone anchored” OR “temporary anchorage device” OR TAD OR implant* OR screw* OR hybrid)
Web of Science	(ALL=(surg* OR “surgically-assisted”)) AND ALL=((palat* OR maxill* OR “upper jaw” OR “upper arch” OR transpalatal) AND (expansion OR expand* OR distract* OR enlargement))) AND ALL=((miniscrew* OR “mini-screw*” OR “micro-screw*” OR “mini-implant*” OR “micro-implant*” OR microimplant* OR “skeletal anchor*” OR “bone-borne” OR “bone borne” OR “bone-anchored” OR “bone anchored” OR “temporary anchorage device” OR TAD OR implant* OR screw* OR hybrid) limit to: Dentistry Oral Surgery Medicine
Scopus	(TITLE-ABS-KEY((surg* OR “surgically-assisted”))) AND (TITLE-ABS-KEY((palat* OR maxill* OR “upper jaw” OR “upper arch” OR transpalatal) AND (expansion OR expand* OR distract* OR enlargement))) AND (TITLE-ABS-KEY (miniscrew* OR “mini-screw*” OR “micro-screw*” OR “mini-implant*” OR “micro-implant*” OR microimplant* OR “skeletal anchor*” OR “bone-borne” OR “bone borne” OR “bone-anchored” OR “bone anchored” OR “temporary anchorage device” OR TAD OR implant* OR screw* OR hybrid))
Advanced search (Cochrane, DARE, Virtual Health Library)	(Title Abstract Keyword) surg* OR “surgically-assisted” AND (palat* OR maxill* OR “upper jaw” OR “upper arch” OR transpalatal) AND (expansion OR expand* OR distract* OR enlargement) AND miniscrew* OR “mini-screw*” OR “micro-screw*” OR “mini-implant*” OR “micro-implant*” OR microimplant* OR “skeletal anchor*” OR “bone-borne” OR “bone borne” OR “bone-anchored” OR “bone anchored” OR “temporary anchorage device” OR TAD OR implant* OR screw* OR hybrid
Manual search terms for trial registries (Clinicaltrials.gov, WHO ICTRP, EU Clinical Trials Register)	Transverse Maxillary Deficiency Malocclusion Crossbite Malocclusion Maxillary Hypoplasia Maxillary Expansion Palatal Expansion SARPE SARME
Manual search terms (Science Direct, Google Scholar, Open Grey, ProQuest)	SARPE bone-borne SARPE bone-borne rapid maxillary expansion surgically assisted bone borne rapid maxillary expansion surgically assisted skeletal anchorage rapid palatal expansion surgically assisted bone borne rapid palatal expansion surgically assisted skeletal anchorage

*: any number of characters or no character.

**Table A2:** Outcomes of studies included in the qualitative synthesis.

Trial ID	Type of Outcomes
Aras et al. (2010) [22]	Nasal area measurements CT: maxillary expansion at canine and molar level
Aras et al. (2017) [23]	Soft and hard tissue variables (SNA, SNB, ANB, among others)
Asscherickx et al. (2016) [24]	Casts: width changes (canine, premolar and molar), angulation changes (premolar and molar), arch perimeter Lateral cephalograms: medio-orbital, nasal cavity, and maxillary widths
Barone et al. (2020) [4]	Interdental and intergingival canine, premolar and molar distances Premolar and molar inclinations
Dowgierd et al. (2018) [25]	Lateral cephalograms: Distances in N-ANS—anterior section, S-PNS—posterior section/Angles in anterior and posterior area (e.g., SNA, SNB, ANB, S-N-ANS, S-N-PNS) CBCT: PA-nose floor, bottom of the base of the nose/distances between first maxillary molars
Hansen et al. (2007) [26]	Transverse expansion of the alveolar process, transverse width and buccal tipping in premolar and molar region
Huizinga et al. (2018) [27]	Lateral expansion in five directions: (1) inferior–anterior, (2) inferior–posterior, (3) superior–posterior of the right maxillary segment, (4) anterior vs. posterior of the anterior part of the maxilla, (5) caudal vs. cranial of the caudal part of the maxilla
Koudstaal et al. (2009) [28]	Palatal depth, width (at canine, premolar, molar level), arch perimeter, SNA, distances (SN to A, SN to PNS), nasal floor, tipping
Kunz et al. (2016) [29]	Amount of expansion and angles of crown tipping from canines through second molars
Landes et al.(2009a) [30]	Skeletal widening, segmental inclination, dental widening, dental tipping, bone resorption at premolars, molars
Landes et al. (2009b) [31]	Skeletal widening, segmental inclination, dental widening, dental tipping, bone resorption at premolars, molars
Laudemann et al. (2009) [32]	Transverse skeletal widening, segmental inclination, bone resorption at premolars, molars
Laudemann et al. (2010) [33]	Transverse skeletal widening, dental widening, dental tipping, attachment loss at frontal teeth, premolars, molars
Laudemann et al. (2011) [34]	Transverse widening, segmental inclination, bone resorption at premolars and molars, dental tipping, pterygoid widening, pterygoid bending, pterygomaxillary inclination
Matteini et al. (2001) [35]	Expansion and expansion ratio for canines, first premolars, first molars, canines
Nada et al. (2012) [36]	Widths: canines, first and second premolars, first and second molars/alveolar expansion at the right, left and anterior segment
Nada et al. (2013) [37]	Maxillary soft tissue changes (upper lip, right and left lateral regions, right and left cheek region) and upper incisor inclination
Nikolaev et al. (2017) [38]	Interapical and intercoronal distances between maxillary canines, premolars and first molars, coronal-apical index
Parhiz et al. (2011) [39]	Angular changes (e.g., SNA, SNB, ANB)
Petrick et al. (2011) [40]	Midpalatal suture: density, width (anterior, median, posterior)
Pinto et al. (2001) [41]	Intercanine, interpremolar, intermolar expansion, expansion ratio, arch periphery gain, angulation of first molars and first premolars
Ploder et al. (2021) [42]	Interdental expansion measurements at tooth and bone levels (canines, first and second premolars, first and second molars)
Seeberger et al. (2015) [43]	Nasal floor expansion (at four different levels) Premolars and molars: crown width, apex width, left and right angle
Tausche et al. (2007) [44]	Transverse expansion at coronal and apical level (central incisors, canines, first premolars, first molars) Transverse expansion at the alveolar crest level (first premolars, first molars) Changes in skeletal structures (orbits, piriform, ANS, PNS, Points A and B, zygomaxillary suture)
Wallner et al. (2022) [45]	Nasal soft tissue variables, nasal skeletal expansion Skeletal maxillary variables (veritcal palate height at first molars, transverse width at canines and first molars)
Xi et al. (2017) [46]	Dental show, mandibular plane angle, occlusal plane angle, vertical position of the maxilla, vertical chin position, anterior maxillary width, posterior maxillary width, horizontal chin position
Zandi et al. (2014) [47]	Skeletal and dental changes: First premolar and first molar region: nasal floor width, palatal bone, interdental root distance, interdental cusp distance
