# Supplementary material for: Dental, Skeletal, and Soft Tissue Changes after Bone-Borne Surgically Assisted Rapid Maxillary Expansion: A Systematic Review and Meta-Analysis
Source: Dent J (Basel). 2023 Jun 1;11(6):143. doi: 10.3390/dj11060143 (PMC10297662; doi:10.3390/dj11060143)
Supplement: Supplementary file 1 [file dentistry-11-00143-s001.zip › dentistry-2316116-supplementary.pdf]

SUPPLEMENTARY MATERIAL

Table 1. Detailed characteristics of studies included in the qualitative synthesis.

Trial ID	Study design	Intervention	Pterygomaxillary disjunction (PMD)	Distractor	Activation time	Number of patients	Mean age	Sex (M/F)	Malocclusion	Measuring method	Timepoints
**Aras et al. (2010)**	prospective cohort	SARME - TPD (disjunction of zygomaticoalveolar crest, anterior surface of the maxillary sinus, and anterior nasal spinal, respectively, in lateral, anterior, and median osteotomies)	no	Transpalatal Distractor, SurgiTec NV (Bruges, Belgium)	1 mm/day (7 - 10 days)	11	27.3	5/6	maxillary transverse deficiency and partial/near total nasal obstruction	CT	T1 (baseline) T2 (6.7 ± 2.3 months after surgical intervention, at expander removal) T3 (12 - 15 months after expander removal)
**Aras et al. (2017)**	retrospective cohort	SARME - TPD (horizontal osteotomies from the piriform aperture to the pterygomaxillary suture, additional vertical midline osteotomy between the upper central incisors)	no	Transpalatal Distractor, SurgiTec NV (Bruges, Belgium)	1 mm/day (7 - 10 days)	16	27.4	9/7	posterior crossbite	lateral cephalometric radiographs	T1 (baseline) T2 (after expansion - before any further ortho treatment)
**Asscherickx et al. (2016)**	prospective cohort	SARME (standard corticotomies of the anterior, lateral, and median bony supports of the maxilla and midpalatal suture separation)	no	Transpalatal Distractor, SurgiTec NV (Bruges, Belgium)	1 mm/3 days (8 - 22 days)	21	26.5	6/15	maxillary transverse deficiency	study casts, posteroanterior cephalograms	T1 (baseline) T2 (end of expansion) T3 (10 weeks after T2)
**Barone et al. (2020)**	prospective cohort	SARME (LeFort I and intermaxillary suture osteotomy, separation of the midline and a nasal septum osteotomy)	no	RPE® expander (KLS Martin, Tuttligen, Germany)	1 mm/day	12	NA	NA	maxillary mandibular transverse discrepancy > 5 mm	virtual study models from dental casts	T1 (baseline) T2 (1 year post-operatively)
**Dowgierd et al. (2018)**	retrospective cohort	SARME (Le Fort I, through the posterior maxillary surface, the zygomatic alveolar crest, anterior wall of the maxillary sinus and edge of the piriform aperture)	yes	Titamed Smile Distractor	approx. 0.6 mm/day	78	16.86	NA	maxillary narrowing with a deficit of the transverse dimension > 4 mm	CBCT, lateral cephalograms	T1 (baseline) T2 (3 months post distraction)
**Haas Junior et al. (2022)**	prospective cohort	SARME (four minimally invasive osteotomies, one subspinal, one vertical midline extended to the nasal floor and two horizontal lateral osteotomies)	no	MARPE expander with four bicortical miniscrews	2 turns/day until appearance of an interincisal diastema, then 1 turn/day until required expansion	11	38.89	3/8	established diagnosis of transverse maxillary deficiency	superimposed CBCT images	T1 (baseline) T2 (after expansion - before any further ortho treatment)
**Hansen et al. (2007)**	prospective cohort	SARME (bilateral osteotomy of the maxillary sinus, pre-formation of the premaxilla above the central incisors)	yes	Dresden distractor	0.96 mm/day (7 days)	12	25.3	NA	serious transverse maxillary constriction	CT	T1 (baseline) T2 (post expansion)
**Huizinga et al. (2018)**	retrospective cohort	SARME (corticotomies of all four bony supports of the maxilla)	yes	TPD (Classic or All-in-one, Surgi-Tec, Sint-Denijs-Westrem, Belgium)	0.33 - 0.66 mm/day	20	24.5	12/8	transversal maxillary hypoplasia	CBCT	T1 (baseline) T2 (post expansion)
**Koudstaal et al. (2009)**	RCT	SARME (LeFort I)	no	Transpalatal Distractor, SurgiTec NV	1 mm/day	23 (25 randomized)	33	10/15	transverse maxillary hypoplasia	lateral and PA cephalograms, casts	T1 (baseline) T2 (post expansion) T3 (12 months

				(Bruges, Belgiumor RPE® expander (KLS Martin, Tuttligen, Germany)							after treatment)
**Kunz et al. (2016)**	retrospective cohort	SARME (subtotal Le Fort I)	yes (partial separation of pterygoid process)	Transpalatal Distractor, SurgiTec NV (Bruges, Belgium or RPE® expander (KLS Martin, Tuttligen, Germany)	0.66 mm/day (2 - 3 weeks)	16	26.5	6-10	pronounced transverse deficit of the maxilla combined with uni- or bilateral posterior crossbite	3D models dental casts	T1 (baseline) T2 (5.6 ± 3.5 months post distraction)
**Landes et al.(2009a)**	retrospective and prospective cohort	SARME (Tripartite or bipartite osteotomies)	yes/no	MWD (Normed, Tuttlingen, Germany) or TPD (Surgi-Tec, Bruges, Belgium)	0.5 - 0.6 mm/day	8 (bipartite) 11 (tripartite)	NA	NA	transverse maxillary compression, dental crowding, lack of adequate dentoalveolar basis	3D-CT scans	T1 (baseline) T2 (post expansion)
**Landes et al.(2009b)**	retrospective and prospective cohort	SARME (Tripartite or bipartite osteotomies)	yes/no	MWD (Normed, Tuttlingen, Germany) or TPD (Surgi-Tec, Bruges, Belgium)	0.5 - 0.6 mm/day	24 (50)	NA	NA	transverse maxillary compression, dental crowding, lack of adequate dentoalveolar basis	3D-CT scans	T1 (baseline) T2 (post expansion)
**Laudemann et al. (2009)**	retrospective and prospective cohort	SARME (LeFort I, tripartite or bipartite osteotomies)	yes/no	MWD (Normed, Tuttlingen, Germany) or TPD (Surgi-Tec, Bruges, Belgium)	0.5 - 0.6 mm/day	24	NA	NA	transverse maxillary compression, dental crowding, or lack of dentoalveolar basis	3D-CT scans	T1 (baseline) T2 (4 - 26 weeks post expansion)
**Laudemann et al. (2010)**	retrospective cohort	SARME (LeFort I, tripartite or bipartite osteotomies)	yes/no	MWD (Normed, Tuttlingen, Germany) or TPD (Surgi-Tec, Bruges, Belgium)	0.5 - 0.6 mm/day	18	NA	NA	insufficient dentoalveolar basis and a transverse maxillomandibular deficit of > 5 mm	3D scanned cast models	T1 (baseline) T2 (20.5 ± 1.34 months post-expansion)
**Laudemann et al. (2011)**	retrospective and prospective cohort	SARME (LeFort I, tripartite or bipartite osteotomies)	yes/no	MWD (Normed, Tuttlingen, Germany) or TPD (Surgi-Tec, Bruges, Belgium)	0.5 - 0.6 mm/day	25	NA	NA	transverse maxillary deficiency, dental crowding or lack of dentoalveolar basis	3D-CT scans	T1 (baseline T2 (2.87 ± 1.59 months post-expansion)
**Matteini et al. (2001)**	prospective cohort	SARME (standard osteotomies)	yes	TPD	0.33 mm/day	20	20	8/12	transverse maxillary deficiency (lateroposterior crossbite)	Models	T1 (before surgery) T2 (post expansion, 2 - 3 weeks later)
**Nada et al. (2012)**	prospective cohort	SARME (LeFort I, additional midline osteotomy)	yes	TPD (Surgi-Tec, Bruges, Belgium)	1 mm/day	17	29.4	NA	skeletal transverse maxillary deficiency combined with another skeletal discrepancy that required orthognathic surgical intervention	3D CBCT models	T1 (baseline), T2 (22 ± 7 months after completion of pre-surgical orthodontic treatment and prior to second orthognathic intervention)
**Nada et al. (2013)**	prospective cohort	SARME (LeFort I, additional midline osteotomy)	yes	TPD (Surgi-Tec, Bruges, Belgium)	1 mm/day	15	30	7/8	developmental deformity, absence of more than four teeth in the posterior maxillary arch, and lips not being in rest position during the CBCT scan	3D CBCT models	T1 (baseline) T2 (22 ± 7 months after completion of pre-surgical orthodontic treatment)
**Nikolaev et al. (2017)**	retrospective cohort	SARME	NA	NA	NA	21	NA	NA	NA	CBCT	T1 (baseline) T2 (day of the expander removal)
**Parhiz et al. (2011)**	retrospective cohort	SARME (Mid-palatal osteotomy with Le Fort I osteotomy and nasal disjunction)	yes	TPD (SurgiTec, Bruges, Belgium)	0.33 mm/day	50	26	20/30	transverse maxillary deficiency	posteroanterior (PA) and lateral cephalograms, panoramic and periapical radiographs, intraoral and extraoral photographs, study models	T1 (baseline) T2 (20 ± 9 months)

**Petrick et al. (2011)**	prospective cohort	SARME (lateral osteotomy)	no	Dresden Distractor (DD; ITU, Dresden, Germany)	1 mm/day (8 days)	16	24.5	7/9	transverse maxillary deficiency	CT	T1 (baseline) T2 (on average 7.01 months after SARME)
**Pinto et al. (2001)**	prospective cohort	SARME (osteotomies)	no	TPD (Surgi-Tec, Bruges, Belgium)	0.33 mm/day	20	21.5	9/11	NA	Digital photographs of the models (dental casts)	T1 (baseline) T2 (end of expansion)
**Ploder et al. (2021)**	retrospective cohort	SARME (corticotomies of the anterior, lateral and medial bony supports of the maxilla)	yes	TPD (Surgi-Tec, Bruges, Belgium) or OMI appliance (Micro-4 Hyrax appliance (MICRO4), Tiger Dental, Bregenz, Austria)	TPD: 1 mm/day (12.6 ± 5.8 days) OMI: 0.51 mm/day (12.5 ± 1.3 days)	12 (TPD) 13 (OMI)	TPD: 24.8 OMI: 36.1	TPD: 5/7 OMI: 4/9	transverse maxillary deficiency (dental crossbite, crowding, constricted maxillary arch, maxillary width discrepancy > 5 mm)	Cast models Panoramic radiographs	T1 (before surgery, 1-7 days) T2 (after consolidation period, 8- 10 weeks) T3 (1 year after surgery, range 10-14 months)
**Seeberger et al. (2015)**	prospective cohort	SARME (LeFort I, including osteotomy of the pterygoid process)	yes	Titamed Uni-Smile Distractor (Wervik, Belgium)	0.5 mm/day	19	22	8/11	transverse maxillary deficiency	CBCT	T1 (1 month before) T2 (3 months after surgery)
**Tausche et al. (2007)**	prospective cohort	SARME (bilateral osteotomy of the lateral maxillary sinus walls)	no	DD - Dresden Distractor	over-compensation of 0.5-1 mm (8-10 days)	17	28.8	6/11	severely constricted upper jaw in conjunction with an Angle Class II, Class III or open bite malocclusion	CT	T1 (before) T2 (6 months after insertion of DD)
**Wallner et al. (2022)**	retrospective cohort	SARME (Le Fort I, midline osteotomy)	yes	Titamed SMILE 3-distractor (Kontich, Belgium)	0.5 mm/day (0.25 mm twice a day) for 14 days	91	20	33/58	transverse maxillary deficiency (loss of at least 5 mm) with closed intermaxillary suture	superimposed CBCT images	T1 (baseline) T2 (approx. 1 year postoperatively)
**Xi et al. (2017)**	retrospective cohort	SARME (Le Fort I, nasal osteotomies, additional midline osteotomy)	yes (only in cases of asymmetric mobility)	TPD (UNI-Smile distractor, Titamed, Kontich, Belgium).	1 mm/day (consolidation period of 8 - 10 weeks)	78 with hyrax group	30.2	22/56	transversal maxillary hypoplasia and mandibular hypoplasia	3D cephalometric reference frame from CBCT	T1 (baseline) T2 (20.3 ± 6.2 months)
**Zandi et al. (2014)**	RCT	SARME (osteotomy of the lateral maxillary wall from the piriform rim to the pterygomaxillary junction, midline osteotomy between the central incisors, and pterygomaxillary disjunction, not including the releasing of the nasal septum)	yes	TPD (SurgiTec, Bruges, Belgium)	0.5 - 0.6 mm/day up to an overexpansion of 2 - 3 mm	15	19.4	5/10	transverse maxillary deficiency	CBCT	T1 (before operation) T2 (immediately after consolidation period)

CBCT: cone beam computed tomography; CT: computed tomography; NA: not available; PA: posteroanterior; RCT: randomized controlled trial

Table 2. Outcomes of studies included in the qualitative synthesis.

Trial ID	Outcomes	*N*	*Mean₀*	*SD₀*	*Mean₁*	*SD₁*	*MD*	*SD*
**Aras et al. (2010)**	*Maxillary canine area*	11						
	Binasal cavity width (CT - mm)		24.32	2.33	29.00	2.93		
	Bimaxillary width (CT - mm)		32.82	8.45	39.41	8.91		
	Bialveolar width (CT - mm)		36.36	3.67	43.73	4.31		
	Maxillary first molar area	11						
	Binasal cavity width (CT - mm)		30.82	2.85	33.55	3.30		
	Bimaxillary width (CT - mm)		52.18	7.98	56.73	6.64		
	Bialveolar width (CT - mm)		48.73	8.45	54.68	8.86		
**Aras et al. (2017)**	Activation average (mm)	16	77.11	4.42	77.74	4.66		
	Hard tissue variables							
	SNA* (°)		77.11	4.42	77.74	4.66		
	SNB* (°)		78.57	4.36	77.93	4.06		
	ANB* (°)		-1.46	3.02	-0.19	3.36		
	ANPg* (°)		-2.85	3.88	-1.79	3.95		
	SNGoGn* (°)		36.15	7.48	37.00	6.91		
	U1-SN (°)		103.61	6.43	102.77	6.17		
	L1-MP (°)		82.91	6.25	82.77	6.23		
	Soft tissue variables							
	Soft tissue facial angle		90.26	8.02	89.64	7.49		
	Nose prominence		16.77	4.12	16.54	3.66		
	Superior sulcus depth		2.16	1.51	2.03	1.41		
	Soft tissue subnasale to H line		2.41	3.08	2.70	2.84		
	Basic upper lip thickness*		16.57	2.50	15.96	2.34		
	Upper lip thickness		13.30	3.07	12.75	2.27		
	H angle		7.94	5.67	8.38	5.29		
	Lower lip to H line		1.46	1.35	1.59	1.69		
	Inferior sulcus to H line		3.43	2.05	3.66	1.89		
	Soft tissue chin thickness		12.35	1.85	12.61	1.78		
	Upper lip strain measurement		3.27	2.31	3.21	2.22		
	Basic upper lip to Ver P		76.22	7.72	76.03	6.98		
	Upper lip to Ver P		78.81	8.57	78.37	8.01		

**Asscherickx et al. (2016)**	Activation average (mm)	21					6	
	Intercanine width (mm)		20.4	2.71	27.0	2.77		
	Intercanine width (mm) (T3)				26.1	2.99		
	Interpremolar width (mm)		23.2	2.52	29.3	2.79		
	Interpremolar width (mm) (T3)				28.9	3.45		
	Intermolar width (mm)		29.5	3.88	34.9	4.01		
	Intermolar width (mm) (T3)				34.9	4.01		
	Arch perimeter (mm)		67.5	6.73	34.8	3.69		
	Arch perimeter (mm) (T3)				73.0	6.56		
	Premolar inclination (°)		198.1	12.80	191.5	14.68		
	Premolar inclination (°) (T3)				193.6	15.24		
	Molar inclination (°)		155.9	15.79	147.7	16.51		
	Molar inclination (°) (T3)				145.3	17.26		
	Medio-orbital width (mm)		21.6	1.61	22.4	1.45		
	Medio-orbital width (mm) (T3)				22.5	1.58		
	Nasal cavity width (mm)		30.3	2.08	33.0	2.55		
	Nasal cavity width (mm) (T3)				32.8	2.66		
	Maxillary width (mm)		55.1	3.93	59.9	4.14		
	Maxillary width (mm) (T3)				59.4	3.74		
**Barone et al. (2020)**	Interdental canine distance (mm)	12					2.18	1.30
	Interdental premolar distance (mm)						4.89	3.05
	Interdental molar distance (mm)						3.22	3.56
	Intergingival canine distance (mm)						2.21	1.60
	Intergingival premolar distance (mm)						4.35	2.86
	Intergingival premolar distance (mm)						4.35	2.86
	Premolar inclination (ΔΔ Interdental − Intergingival premolar distance) (mm)						0.545	0.466
	Molar inclination (ΔΔ Interdental − Intergingival molar distance) (mm)						0.555	1.006
**Dowgierd et al. (2018)**	S-N-PNS	78	39.54	4.77	40.75	3.46		
	S-PNS-ANS		113.53	4.95	114.05	5.21		
	S-N-ANS		7.26	5.78	7.05	4.09		
	SN-OCL		16.67	5.91	16.35	4.58		
	ANB		-0.75	4.47	0.48	4.04		

	SNA		82.52	5.69	81.28	4.19		
	SNB		82.05	5.23	81.60	5.06		
	S-PNS		47.42	4.69	47.87	4.48		
	N-ANS		50.72	4.24	51.32	4.45		
	PA-nosefloor		24.49	4.14	25.72	4.08		
	CT_6-6		36.94	3.47	41.77	4.26		
	CT_6-6_palat_plate		31.44	3.06	36.25	3.92		
	CT_palet_H		14.32	2.41	13.38	2.67		
**Haas Junior et al. (2022)**	Posterior maxilla distance	11					3	0.6
	Posterior midpalatal suture distance						1.8	0.3
	Anterior maxilla distance						3.7	0.6
	Anterior midpalatal suture distance						2	0.4
	Posterior alveolar process distance						3.2	0.6
	Anterior alveolar process distance						4.4	0.7
	Posterior dental crown distance						4.3	0.5
	Posterior dental root distance						4.7	0.5
	Angle UR6						0.9	0.6
	Angle UL6						2.3	1.4
**Hansen et al. (2007)**	*Buccal tipping (°)*	12					4.62	3.72
	Premolar right (°)						3.09	4.44
	Premolar left (°)						2.58	4.07
	Molar right (°)						1.13	6.02
	Molar left (°)						8.62	5.17
	Right alveolar crest premolar region (Ectopremolare) (°)						8.22	4.85
	Right alveolar crest premolar region (Endopremolare) (°)						9.66	9.18
	Left alveolar crest premolar region (Ectopremolare) (°)						7.99	6.37
	Left alveolar crest premolar region (Endopremolare) (°)						8.01	3.04
	Right alveolar crest molar region (Ectomolare) (°)						8.05	3.52
	Right alveolar crest molar region (Endomolare) (°)						9.75	6.41
	Left alveolar crest molar region (Ectomolare) (°)						8.74	8.54
	Left alveolar crest molar region (Endomolare) (°)						3.00	1.49

	Midpalatal suture expansion - anterior nasal spine (mm)						0.97	0.92
	Midpalatal suture expansion - posterior nasal spine (mm)						4.24	3.53
	Transverse expansion at A-point						4.62	3.72
	Alveolar process expansion - premolars (mm)						5.55	2.63
	Alveolar process expansion - molars (mm)						4.87	2.44
	Crown expansion - premolars (mm)						6.07	2.97
	Crown expansion - molars (mm)						5.71	2.42
	Root-apex expansion - premolars (mm)						4.28	2.99
	Root-apex expansion - molars (mm)						4.98	2.28
**Huizinga et al. (2018)**	*Lateral expansion in five directions*						MEDIAN	IQR
	Inferior-anterior of the right maxillary segment (mm)						0.36	-1.42, 3.29
	Inferior-posterior of the right maxillary segment (mm)						-0.03	-0.54, 1.74
	Superior-posterior of the right maxillary segment (mm)						0.10	-0.45, 1.46
	Anterior vs. posterior of the anterior part of the maxilla (mm)						-0.53	-1.40, 1.13
	Caudal vs. cranial of the caudal part of the maxilla (mm)						1.51	0.69, 1.93
**Koudstaal et al. (2009)**	Width - Canine (mm) (T1-T2)	23	28.9	4.2	34.9	5.7		
	Width - Canine (mm) (T3)				33.6	3.6		
	Width - Premolar (mm) (T1-T2)		35.2	3.4	42.2	3.8		
	Width - Premolar (mm) (T3)				42.3	3		
	Width - Molar (mm) (T1-T2)		47.1	3.5	52.3	3.7		
	Width - Molar (mm) (T3)				51.7	3.6		
	Arch perimeter (mm) (T1-T2)		64.2	8.9	71.5	8.8		
	Arch perimeter (mm) (T3)				70	7.2		
	Palatal depth premolar (mm) (T1-T3)		20.3	4.6	20.2	4		
	Palatal depth molar (mm) (T1-T3)		22.7	2.6	22.3	2.5		
	Palatal width premolar (mm) (T1-T3)		12.2	4	15.1	3.5		
	Palatal width molar (mm) (T1-T3)		16.6	4.7	19.2	4.3		
	SNA (°) (T1-T3)		79.7	4	80.2	4.3		
	Distance SN to A (mm) (T1-T3)		61.7	5.2	62.4	5		
	Distance SN to PNS (mm) (T1-T3)		48	4.8	48.8	4.7		
	Nasal floor (mm) (T1/T2)		17.8	3	20.4	2.3		
	Nasal floor (mm) (T3)				19.2	2.7		
	Maxilla floor (mm) (T1/T2)		58.8	4	62.1	3.8		

	Maxilla floor (mm) (T3)		61.4	3.8		
	Maxillary tipping (mm) (T1/T2)				0.7	2.6
	Maxillary tipping (mm) (T1/T3)				1.2	2.1
**Kunz et al. (2016)**	Transverse distance canines (mm)	16			4.43	2.21
	Transverse distance first premolars (mm)				4.56	2.27
	Transverse distance second premolars (mm)				4.18	1.77
	Transverse distance first molars (mm)				3.53	1.83
	Transverse distance second molars (mm)				2.72	1.19
	Tooth inclination right canine (°)				-0.57	5.20
	Tooth inclination left canine (°)				-1.12	4.89
	Tooth inclination right first premolar (°)				−1.17	6.00
	Tooth inclination left first premolar (°)				0.17	3.52
	Tooth inclination right second premolar (°)				-1.33	3.76
	Tooth inclination left second premolar (°)				-1.08	4.15
	Tooth inclination right first molar (°)				-1.47	4.25
	Tooth inclination left first molar (°)				-0.73	4.07
	Tooth inclination right second molar (°)				-2.22	3.62
	Tooth inclination left second molar (°)				-0.08	3.02
**Landes et al.(2009a)**	Skeletal widening: HP.PA.4.7 - Bipartite (mm)	8			-2.73	2.24
	Skeletal widening: HP.PA.4.7 - Tripartite (mm)	11			-4.4	3.59
	Skeletal widening: BAC.LAC.4.7 - Bipartite (mm)	8			-3.01	2.14
	Skeletal widening: BAC.LAC.4.7 - Tripartite (mm)	11			-3.98	2.39
	Segmental Inclination: HP.PA.BAC.LAC.4.7 - Bipartite (mm)	8			-0.60	2.95
	Segmental Inclination: HP.PA.BAC.LAC.4.7 - Tripartite (mm)	11			4.44	2.38
	Dental widening: DAE.DAI.4.7 - Bipartite (mm)	8			-3.76	2.18
	Dental widening: DAE.DAI.4.7 - Tripartite (mm)	11			-4.5	4.42
	Dental widening: DAE'.DAI'.4.7 - Bipartite (mm)	8			-4.05	2.99
	Dental widening: DAE'.DAI'.4.7 - Tripartite (mm)	11			-5.08	5.29
	Dental tipping: HP.PA.DAE.DAI.4.7 -Biapartite (mm)	8			-1.18	3.03
	Dental tipping: HP.PA.DAE.DAI.4.7 - Triapartite (mm)	11			-0.37	5.97
	Bone resorption: premolar - Biapartite (mm)	8			-0.41	0.77
	Bone resorption: premolar - Triapartite (mm)	11			-0.13	0.93
	Bone resorption: molar - Biapartite (mm)	8			-0.38	0.4
	Bone resorption: molar - Triapartite (mm)	11			-0.31	0.27

**Landes et al. (2009b)**	*Transverse Skeletal Widening of the Maxilla*			
	HP.PA4 (mm)	21	6.51	3.19
	BAC.LAC4 (mm)	22	7.16	2.91
	HP.PA5 (mm)	21	3.38	1.75
	BAC.LAC5 (mm)	24	6.39	2.87
	HP.PA6 (mm)	19	3.19	1.87
	BAC.LAC6 (mm)	23	5.07	1.63
	HP.PA7 (mm)	21	2.6	1.7
	BAC.LAC7 (mm)	23	3.42	2.31
	HP.PA.4.7 (mm)	19	-3.7	3.13
	BAC.LAC.4.7 (mm)	21	-3.51	2.28
	Transverse Dental Widening of the Maxilla			
	DA.E'.DA.I'4 (mm)	13	7.30	2.49
	DA.E.DA.I4 (mm)	14	7.51	3.3
	DA.E'.DA.I'5 (mm)	14	6.68	3.21
	DA.E.DA.I5 (mm)	10	6.89	3.44
	DA.E'.DA.I'6 (mm)	13	6.84	1.97
	DA.E.DA.I6 (mm)	12	7.12	2.29
	DA.E'.DA.I'7 (mm)	12	3.24	1.63
	DA.E.DA.I7 (mm)	12	3.42	2.31
	DA.E'.DA.I'.4.7 (mm)	11	-4.16	3.44
	DA.E.DA.I.4.7 (mm)	10	-4.67	2.28
	DA.E'.DA.I'4 (mm)	13	7.30	2.49
	Segmental Inclination and Dental Tipping			
	HP.PA.BAC.LAC.4	21	0.65	1.37
	HP.PA.BAC.LAC.5	21	2.85	2.29
	HP.PA.BAC.LAC.6	19	1.92	1.39
	HP.PA.BAC.LAC.7	21	0.41	1.58
	HP.PA.BAC.LAC.4.7	19	-0.11	2.33
	HP.PA.DA.E.DA.I.4	14	0.78	2.43
	HP.PA.DA.E.DA.I.5	10	3.94	2.14
	HP.PA.DA.E.DA.I.6	12	4.02	2.22
	HP.PA.DA.E.DA.I.7	12	0.68	3.0
	HP.PA.DA.E.DA.I.4.7	10	-0.69	4.81

	HP.PA.BAC.LAC.4			
	Vestibular Bone Resorption	22	-0.85	2.57
	TA.BAC 4(mm)	22	-0.02	0.13
	BTA.E 4(mm)	22	0.21	1.99
	LTA.HP 4(mm)	24	-0.88	2.15
	TA.BAC 5(mm)	24	-0.19	1.03
	BTA.E 5(mm)	24	0.08	0.72
	LTA.HP 5(mm)	24	0	0.3
	TA.BAC 6(mm)	24	-0.59	0.59
	BTA.E 6(mm)	24	-0.05	0.6
	LTA.HP 6(mm)	24	-0.89	1.53
	TA.BAC 7(mm)	24	-0.39	0.9
	BTA.E 7(mm)	24	-0.18	0.51
**Laudemann et al. (2009)**	BLP.BMP (transverse widening at pterygoid level)	24	-3.3	3.7
	Angle l-l (°) (lateral bending of the pterygoid processes)	24	7.0	18.8
	Transverse widening HP.PA.4.7 (mm) (with PMD)	11	−4.50	3.91
	Transverse widening HP.PA.4.7 (mm)	13	−2.61	2.43
	Transverse widening BAC.LAC.4.7 (mm) (with PMD)	11	−3.00	2.44
	Transverse widening BAC.LAC.4.7 (mm)	13	−3.52	2.69
	Segmental inclination HP.PA.BAC.LAC.4.7 (mm) (with PMD)	11	1.50	2.86
	Segmental inclination HP.PA.BAC.LAC.4.7 (mm)	13	−0.91	1.89
	Transverse widening (dental arch to pterygoid level) BLP.BMP. HP.PA.BAC.LAC6.7 (mm) (with PMD)	11	−1.77	4.14
	Transverse widening (dental arch to pterygoid level) BLP.BMP. HP.PA.BAC.LAC6.7 (mm)	13	0.57	2.97
	Bone resorption premolars (mm) (with PMD)	11	0.29	0.53
	Bone resorption premolars (mm)	13	−0.74	0.77
	Bone resorption molars (mm) (with PMD)	11	−0.36	0.36
	Bone resorption molars (mm)	13	−0.34	0.33
**Laudemann et al. (2010)**	Transverse Skeletal Widening GM.3.7 (mm) (gingival margin)	7	1.61	1.76
	Transverse Skeletal Widening CT.3.7 (mm) (cusp tips)	13	0.99	3.28
	Intercanine GM.CT (mm) (dental tipping)	12	−0.21	0.95
	1st premolar GM.CT (mm)	10	−0.06	1.27
	2nd premolar GM.CT (mm)	10	0.54	1.15

	1st molar GM.CT (mm)	9	0.22	1.01
	2nd molar GM.CT (mm)	8	0.97	0.64
	Ccl 1 (Clinical crown length - attachment loss)	17	0.4	1.14
	Ccl 2	12	0.78	0.85
	Ccl 3	14	0.51	0.75
	Ccl 4	10	0.44	0.83
	Ccl 5	10	0.40	0.65
	Ccl 6	9	−0.12	0.55
	Ccl 7	8	0.23	0.62
**Laudemann et al. (2011)**		25 Overall in BB group		
	Transverse Widening (HP.PA.4.7)(<20 years old) (with PMD)		-4.72	5.34
	Transverse Widening (HP.PA.4.7) (>20) (with PMD)		-4.03	1.73
	Transverse Widening (HP.PA.4.7)(<20)		-2.77	2.09
	Transverse Widening (HP.PA.4.7)(>20)		-3.01	2.83
	Transverse Widening (BAC.LAC.4.7) (<20) (with PMD)		-2.50	3.07
	Transverse Widening (BAC.LAC.4.7)(>20) (with PMD)		-3.18	2.16
	Transverse Widening (BAC.LAC.4.7)(<20)		-3.58	2.32
	Transverse Widening (BAC.LAC.4.7)(>20)		-4.48	1.33
	Segmental Inclination (HP.PA.BAC.LAC.4.7)(<20) (with PMD)		1.81	2.78
	Segmental Inclination (HP.PA.BAC.LAC.4.7)(>20) (with PMD)		0.34	1.62
	Segmental Inclination (HP.PA.BAC.LAC.4.7)(<20)		-0.81	2.18
	Segmental Inclination (HP.PA.BAC.LAC.4.7)(>20)		-1.47	2.08
	Bone Resorption: Premolars (<20) (with PMD)		0.42	0.41
	Bone Resorption: Premolars (>20) (with PMD)		0.08	0.24
	Bone Resorption: Premolars (<20)		-0.49	0.41
	Bone Resorption: Premolars (>20)		-0.5	0.67
	Bone Resorption: Molars (<20) (with PMD)		-0.46	0.50
	Bone Resorption: Molars (>20) (with PMD)		-0.42	0.56
	Bone Resorption: Molars(<20)		-0.45	0.49
	Bone Resorption: Molars(>20)		-0.47	0.15
	Dental Tipping (HP.PA.DA.E.DA.I.4.7)(<20) (with PMD)		1.13	2.28
	Dental Tipping (HP.PA.DA.E.DA.I.4.7)(>20) (with PMD)		0.90	0.22
	Dental Tipping (HP.PA.DA.E.DA.I.4.7)(<20)		-1.73	2.05

	Dental Tipping (HP.PA.DA.E.DA.I.4.7)(>20)		0.53	2.10
	Pterygoid Widening (BLP.BMP)(<20) (with PMD)		-2.91	4.72
	Pterygoid Widening (BLP.BMP)(>20) (with PMD)		0.81	3.33
	Pterygoid Widening (BLP.BMP)(<20)		0.31	2.64
	Pterygoid Widening (BLP.BMP)(>20)		-0.16	5.95
	Pterygoid Bending (Angle 1-1)(<20) (with PMD)		5.08	23.06
	Pterygoid Bending (Angle 1-1)(>20) (with PMD)		4.83	8.09
	Pterygoid Bending (Angle 1-1)(<20)		7.17	27.65
	Pterygoid Bending (Angle 1-1)(>20)		-2.13	20.00
	Pterygomaxillary Inclination(BLP.BMP.HP.PA.BAC.LAC.6.7)(<20) (with PMD)		-3.04	2.88
	Pterygomaxillary Inclination(BLP.BMP.HP.PA.BAC.LAC.6.7)(>20) (with PMD)		-2.42	0.97
	Pterygomaxillary Inclination(BLP.BMP.HP.PA.BAC.LAC.6.7)(<20)		-0.74	3.36
	Pterygomaxillary Inclination(BLP.BMP.HP.PA.BAC.LAC.6.7)(>20)		0.99	4.80
	Transverse Widening (BAC.LAC.4.7)(2-segment osteotomy) (with PMD)		-3.01	2.80
	Transverse Widening (BAC.LAC.4.7)(3-segment osteotomy) (with PMD)		-2.69	2.68
	Transverse Widening (BAC.LAC.4.7)(2S)		-2.99	1.87
	Transverse Widening (BAC.LAC.4.7)(3S)		-5.13	5.95
	Pterygoid Bending (Angle 1-1)(2S) (with PMD)		11.50	17.68
	Pterygoid Bending (Angle 1-1)(3S) (with PMD)		0.29	15.11
	Pterygoid Bending (Angle 1-1)(2S)		18.79	24.93
	Pterygoid Bending (Angle 1-1)(3S)		-12.58	11.38
	Pterygomaxillary Inclination (BLP.BMP.HP.PA.BAC.LAC.6.7)(2S) (with PMD)		-2.23	1.33
	Pterygomaxillary Inclination (BLP.BMP.HP.PA.BAC.LAC.6.7)(3S) (with PMD)		-2.99	2.58
	Pterygomaxillary Inclination (BLP.BMP.HP.PA.BAC.LAC.6.7)(2S)		0.56	4.13
	Pterygomaxillary Inclination (BLP.BMP.HP.PA.BAC.LAC.6.7)(3S)		-1.27	3.07
**Matteini et al. (2001)**	expansion canines (%)	20	29.9	14.1
	expansion 1st premolars (%)		28.3	11.6
	expansion 1st molars (%)		20.8	7.2
	expansion ratio canine level (%)		22.4	
	expansion ration 1st premolar level (%)		21.9	
	expansion ratio 1st molar level (%)		20.8	

**Nada et al. (2012)**	*Interocclusal*							
	Width at canines (mm)	17	30.29	3.1	36.05	2.3		
	Width at first premolars (mm)		36.77	2.4	43.02	1.7		
	Width at second premolars(mm)		40.79	2.3	47.37	3.1		
	Width at first molars (mm)		45.01	4.3	52.15	3.4		
	Width at second molars (mm)		52.12	4.1	56.71	3		
	Interappical							
	Width first premolars Ap-Ap`(mm)		31.32	4.2	36.56	4.2		
	Width first molars Ap-Ap`(mm)		31.77	3.54	36.37	2.9		
	Intercoronal							
	Width at first premolars Cb-Cb`(mm)		36.58	3.1	43.54	1.8		
	Width at first molars Cb-Cb`(mm)		46.83	4	53.6	3.17		
	Alveolar expansion Right segment (mm)						1.91	0.97
	Alveolar expansion Left segment (mm)						1.69	0.68
	Alveolar expansion Anterior segment (mm)						-1.35	1.53
**Nada et al. (2013)**	*Soft tissues*							
	L-mid (mm) (region of upper lip)	15					−1.6	1.9
	L-right (mm)						−0.45	2.3
	L-left (mm)						−0.48	1.8
	C-right (mm) (cheek region posterior to the angle of the mouth)						1.48	1.6
	C-left (mm)						0.82	1.6
	Hard tissues							
	B-mid (mm) (anterior maxillary segment)						−1.12	1.5
	B-right (mm) (posterior maxillary segment)						1.97	0.9
	B-left (mm) (posterior maxillary segment)						1.82	0.9
	U1/pp (°)		112.01	7.1	102.72	7.6		
**Nikolaev et al. (2017)**	Interapical distance canines (mm)	21					2.6	0.3
	Interapical distance 1st premolars (mm)						3.1	0.4
	Interapical distance 2nd premolars						2.3	0.3
	Interapical distance molars (mm)						2.3	0.3
	Intercoronal distance canines (mm)						3.9	0.4
	Intercoronal distance 1st premolars (mm)						4.8	0.5

	Intercoronal distance 2nd premolars(mm)						4.7	0.5
	Intercoronal distance molars(mm)						4.1	0.4
**Parhiz et al. (2011)**	U1-SN	50					-4.82	8.94
	U1-PP						-3.86	8.56
	PP-SN						0.94	2.13
	PP-Mand.						-0.22	3.38
	SNA						1.60	2.57
	SNB						0.46	2.61
	ANB						1.06	2.00
	SN-Mand.						0.66	3.03
**Petrick et al. (2011)**	Midpalatal suture density - anterior (HU)	16	950	167	16	459		
	Midpalatal suture density - median (HU)		845	125	16	452		
	Midpalatal suture density - posterior (HU)		1046	103	16	787		
	Midpalatal suture width - anterior (mm)		3.41	1.74	5.71	2.57		
	Midpalatal suture width - median (mm)		2.79	1.55	3.90	2.08		
	Midpalatal suture width - posterior (mm)		2.32	1.06	2.57	1.13		
**Pinto et al. (2001)**	Expansion canines (%)	20					35.7	17
	Expansion first premolars (%)						31.7	14
	Expansion first molars (%)						20.4	8.7
	Arch periphery gain after expansion						10.5	4.6
	Angulation of first premolars (°)						-8.3	9.6
	Angulation of first molars (°)						0.9	9.9
**Ploder et al. (2021)**	*Tooth level - TPD*	12						
	Overall expansion tooth level (T1-T2) (mm)						5.22	1.72
	Overall expansion tooth level (T2-T3) (mm)						0.76	1.37
	Expansion canines tooth level (T1-T2) (mm)						4.76	3.00
	Expansion canines tooth level (T2-T3) (mm)						1.33	1.25
	Expansion 1st premolars tooth level (T1-T2) (mm)						4.82	1.22
	Expansion 1st premolars tooth level (T2-T3) (mm)						0.94	1.67
	Expansion 2nd premolars tooth level (T1-T2) (mm)						6.82	1.86
	Expansion 2nd premolars tooth level (T2-T3) (mm)						0.13	2.51
	Expansion 1st molars tooth level (T1-T2) (mm)						4.91	2.64
	Expansion 1st molars tooth level (T2-T3) (mm)						0.06	1.63

Expansion 2nd molars tooth level (T1-T2) (mm)		4.47	2.71
Expansion 2nd molars tooth level (T2-T3) (mm)		0.85	1.42
Tooth level - OMI	13		
Overall expansion tooth level (T1-T2) (mm)		4.66	2.03
Overall expansion tooth level (T2-T3) (mm)		0.71	0.96
Expansion canines tooth level (T1-T2) (mm)		4.38	1.57
Expansion canines tooth level (T2-T3) (mm)		0.85	1.59
Expansion 1st premolars tooth level (T1-T2) (mm)		4.91	2.29
Expansion 1st premolars tooth level (T2-T3) (mm)		0.86	0.83
Expansion 2nd premolars tooth level (T1-T2) (mm)		5.19	2.57
Expansion 2nd premolars tooth level (T2-T3) (mm)		0.97	1.61
Expansion 1st molars tooth level (T1-T2) (mm)		4.89	3.08
Expansion 1st molars tooth level (T2-T3) (mm)		0.5	1.55
Expansion 2nd molars tooth level (T1-T2) (mm)		3.69	2.34
Expansion 2nd molars tooth level (T2-T3) (mm)		0.36	1.47
Bone level - TPD	12		
Overall expansion bone level (T1-T2) (mm)		4.66	2.03
Overall expansion bone level (T2-T3) (mm)		0.71	0.96
Expansion canines bone level (T1-T2) (mm)		4.38	1.57
Expansion canines bone level (T2-T3) (mm)		0.85	1.59
Expansion 1st premolars bone level (T1-T2) (mm)		4.91	2.29
Expansion 1st premolars bone level (T2-T3) (mm)		0.86	0.83
Expansion 2nd premolars bone level (T1-T2) (mm)		5.19	2.57
Expansion 2nd premolars bone level (T2-T3) (mm)		0.97	1.61
Expansion 1st molars bone level (T1-T2) (mm)		4.89	3.08
Expansion 1st molars bone level (T2-T3) (mm)		0.5	1.55
Expansion 2nd molars bone level (T1-T2) (mm)		3.69	2.34
Expansion 2nd molars bone level (T2-T3) (mm)		0.36	1.47
Bone level - OMI	13		
Overall expansion bone level (T1-T2) (mm)		3.51	1.66
Overall expansion bone level (T2-T3) (mm)		0.12	1.56
Expansion canines bone level (T1-T2) (mm)		3.99	1.99
Expansion canines bone level (T2-T3) (mm)		0.00	1.86
Expansion 1st premolars bone level (T1-T2) (mm)		4.37	2.17
Expansion 1st premolars bone level (T2-T3) (mm)		0.09	1.49

	Expansion 2nd premolars bone level (T1-T2) (mm)		3.35	1.50
	Expansion 2nd premolars bone level (T2-T3) (mm)		-0.06	1.98
	Expansion 1st molars bone level (T1-T2) (mm)		3.20	1.64
	Expansion 1st molars bone level (T2-T3) (mm)		0.28	1.48
	Expansion 2nd molars bone level (T1-T2) (mm)		2.46	1.69
	Expansion 2nd molars bone level (T2-T3) (mm)		0.04	1.83
**Seeberger et al. (2015)**		19	MEDIAN	IQR
	Expansion of maxilla (mm)		5.0	
	Nasal isthmus 1 (mm) (anterior to posterior)		3.0	3.2
	Nasal floor 2 (mm)		1.5	3.4
	Nasal floor 3 (mm)		1.1	1.8
	Nasal floor 4 (mm)		0.9	3.1
	Premolar crown width (mm)		4.6	3.4
	Premolar apex width (mm)		3.3	3.1
	Premolar angle right (°)		-1.49	6.96
	Premolar angle left (°)		-2.61	3.01
	Molar crown width (mm)		3.40	2.4
	Molar apex width (mm)		3.20	2.8
	Molar angle right (°)		-3.44	6.78
	Molar angle left (°)		-1.13	5.72
**Tausche et al. (2007)**	*Transverse expansion - Teeth*	10		
	Maxillary central incisors (diastema) (mm)		4.57	2.13
	Maxillary canines (mm)		5.59	2.75
	Maxillary first premolars (mm)		6.72	2.58
	Maxillary first molars (mm)		6.44	1.92
	Apices of maxillary central incisors (mm)		3.25	2.89
	Apices of maxillary canines (mm)		5.72	3.39
	Buccal apices of first premolars (mm)		5.79	2.65
	Mesiobuccal apices of maxillary first molars (mm)		6.53	2.07
	Transverse expansion – Alveolar crest			
	Ectoprämolare (mm)		7.27	2.75
	Endoprämolare (mm)		7.76	3.18

	Ectomolare (mm)						7.15	2.3
	Endomolare (mm)						7.18	1.88
	Transverse expansion – Alveolar crest							
	Lower margins of orbitae (mm)						0.012	0.01
	Piriform (mm)						1.59	0.58
	Anterior nasal spine (mm)						3.91	2.49
	Posterior nasal spine (mm)						1.42	2.03
	Zygomaxillare (mm)						0.11	0.1
	Point A (mm)						4.18	3.03
	Point B (mm)						-0.17	0.2
	Buccal tipping – Teeth							
	Maxillary first premolar, right (°)						3.0	
	Maxillary first premolar, left (°)						3.9	
	Maxillary first molar, right (°)						3.5	
	Maxillary first molar, left (°)						2.5	
	Buccal tipping – Alveolar crest							
	Premolar region, right Ectoprämolare (°)						11.1	
	Premolar region, right Endoprämolare (°)						13.3	
	Premolar region, right average (°)						12.2	
	Premolar region, left Ectoprämolare (°)						10.8	
	Premolar region, left Endoprämolare (°)						9.9	
	Premolar region, left average (°)						10.4	
	Molar region, right Ectomolare (°)						10.6	
	Molar region, right Endomolare (°)						11.8	
	Molar region, right average (°)						11.2	
	Molar region, left Ectomolare (°)						11.8	
	Molar region, left Endomolare (°)						10.2	
	Molar region, left average (°)						11	
**Wallner et al. (2022)**	Alar nasal width (mm)	91	32.9	2.8	34.1	2.9	1.2	1.1
	Alar nasal base width (mm)		30.8	2.6	32.8	2.9	2.1	1.2
	Nose Tip Height (mm)		27.1	2.6	27.0	2.5	-0.1	1.0
	Nasal apertura width (mm)		22.5	1.6	24.1	2.2	1.5	1.6
	Nasal width at first molar (mm)		30.8	2.5	32.6	2.7	1.8	1.2
	Angle of septal deviation (°)		9.0	4.5	8.7	4.4	-0.2	1.6

	Vertical palate height at first molars (mm)		21.6	2.5	21.5	2.6	−0.1	1.0
	Transversal width at canines (mm)		34.5	3.3	39.3	3.4	4.8	1.8
	Transversal width at first molars (mm)		54.1	3.8	58.0	3.6	3.8	1.4
**Xi et al. (2017)**	Dental show (mm)	78 (with hyrax group)					2.5	2.1
	Mandibular plane angle (°)						1.1	1.1
	Occlusal plane angle (°)						4.9	4.7
	Vertical changes at A-point (mm)						1.6	2.3
	Vertical changes at pogonion (mm)						1.8	1.8
	Anterior maxillary expansion (mm)						1.8	1.0
	Posterior maxillary expansion (mm)						2.6	1.8
	Chin advancement (mm)						-1.5	2.2
**Zandi et al. (2014)**	PBW4 - palatal bone width (mm)	15					4.53	2.02
	PBW6 - palatal bone width (mm)						4.33	1.23
	NFW4 - nasal floor width (mm)						1.47	0.52
	NFW6 - nasal floor width (mm)						1.33	0.49
	ICD4 - interdental cusp distance - dental arch (mm)						6.73	2.15
	ICD6 - interdental cusp distance (mm)						6.53	2.67
	IRD4 - interdental root distance (mm)						4.4	1.68
	IRD6 - interdental root distance (mm)						4.5	1.83
